# Causal effect of iron status on lung function: A Mendelian randomization study

**DOI:** 10.3389/fnut.2022.1025212

**Published:** 2022-12-15

**Authors:** Zhimin Yu, Chengkai Xu, Chenggang Fang, Fangfang Zhang

**Affiliations:** ^1^Department of General Medicine and Geriatrics, Zhongnan Hospital of Wuhan University, Wuhan University, Wuhan, China; ^2^Department of Cardiology, Taihe Hospital, Hubei University of Medicine, Shiyan, China; ^3^Department of Pediatrics, Taihe Hospital, Hubei University of Medicine, Shiyan, China

**Keywords:** Mendelian randomization, lung function, FEV1, FVC, iron

## Abstract

**Background:**

The association between systemic iron status and lung function was conflicting in observational studies. We aim to explore the potential causal relationships between iron status and the levels of lung function using the two-sample Mendelian randomization (MR) design.

**Methods:**

Genetic instruments associated with iron status biomarkers were retrieved from the Genetics of Iron Status (GIS) consortium (*N* = 48,972). Summary statistics of these genetic instruments with lung function were extracted from a meta-analysis of UK Biobank and SpiroMeta consortium (*N* = 400,102). The main analyses were performed using the inverse-variance weighted method, and complemented by multiple sensitivity analyses.

**Results:**

Based on conservative genetic instruments, MR analyses showed that genetically predicted higher iron (beta: 0.036 per 1 SD increase, 95% confidence interval (CI): 0.016 to 0.056, *P* = 3.51 × 10^−4^), log10-transformed ferritin (beta: 0.081, 95% CI: 0.047 to 0.116, *P* = 4.11 × 10^−6^), and transferrin saturation (beta: 0.027, 95% CI: 0.015 to 0.038, *P* = 1.09 × 10^−5^) were associated with increased forced expiratory volume in 1 s (FEV1), whereas higher transferrin was associated with decreased FEV1 (beta: −0.036, 95% CI: −0.064 to −0.008, *P* = 0.01). A significant positive association between iron status and forced vital capacity (FVC) was also observed. However, there is no causal association between iron status and FEV1-to-FVC ratio (*P* = 0.10). Similar results were obtained from the liberal instruments analyses and multiple sensitivity analyses.

**Conclusion:**

Our study provided strong evidence to support that higher iron status is causally associated with higher levels of FEV1 and FVC, but has no impact on airway obstruction, confirming iron status as an important target for lung function management.

## Introduction

Chronic obstructive pulmonary disease (COPD) is a common disorder worldwide that has caused heavy disease burden (1). The global prevalence of COPD was estimated at ~174 million cases based on the Global Burden of Disease Study 2015 (2) and the proportion can reach 28% in populations aged 80 years (3). Persistent decline in lung function as assessed by forced expiratory volume in 1 second (FEV1) underlies the pathogenesis of COPD (4). It has been long considered that exposure to particles and gases from tobacco smoking and biomass fuel are the major etiology of lung function decline. However, emerging evidence supports the notion that events early in life affect lung function in adults (5). This fundamentally changed the concept of COPD to show that factors determining the maximal lung capacity attained during development may play important roles in the pathogenesis of the disease (5). Seeking potential risk factors that are involved in lung function will assist in preventing the deterioration of lung function and associated death.

As an essential trace element, iron participates in various aspects of lung diseases, including oxygen delivery, immune response, and oxidative stress (6). Iron has been reported to be critical for effective immune response in defending respiratory tract infection (7). Besides, the early life iron status may determine the maximum lung function by influencing the development of airways and lungs. Current evidence on the association between iron status and lung function is conflicting. An observational study conducted in US women aged 20–49 years in National Health and Nutrition Examination Survey (NHANES) reported that total body iron status was positively associated with FEV1 (8). Similar results were achieved in children aged 10–12 years, where participants with lower iron status have decreased lung function (9). However, these findings contrast with the results of a large cross-sectional study performed in 42,927 Korean men that suggests a negative association between ferritin level and lung function as indicated by FEV1 and forced vital capacity (FVC) (10). In addition, serum ferritin has been associated with increased risk of restrictive respiratory disease in postmenopausal women (11).

Making causal inference from relationships that may be attributable to confounding in these observational studies is challenging. Recently, Mendelian randomization (MR) has emerged as a reliable method to estimate causal association between exposure and outcome by using genetic variants as proxy for exposure (12). Given the non-modifiable and unconfounded nature of genetic instruments, MR study can largely overcome these issues in traditional observational studies. The MR design has been previously adopted to study the causal associations of iron status with risk of asthma and lung cancer (13, 14). However, the causal nature between systemic iron status and lung function remains to be investigated. The aim of the present study was to explore the association of genetically instrumented iron status with lung function using the MR approach.

## Methods

### Study design

**Figure 1:**
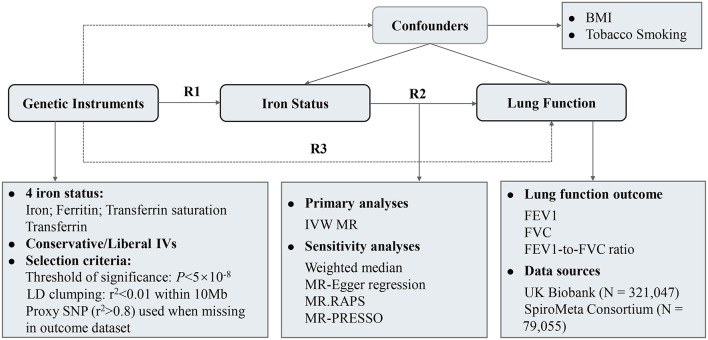
Study design and overview of the MR study. Summary-level GWAS statistics from participants of European descent were used in present study. *R*2 represents the causal effect estimates of iron status on spirometric function, calculated using formula *R*2 = *R*3/*R*1. R1 and R3 denote the direct relations of genetic instruments on iron status and spirometric function (FEV1, FVC, and FEV1/FVC ratio), respectively. BMI, body mass index; FEV1, forced expired volume in 1 s; FVC, forced vital capacity; GWAS, genome-wide association study; IVs, instrumental variables; IVW, inverse variance weighted; LD, linkage disequilibrium.

Figure 1 depicted the overall design of the present study. The causal association between iron status and lung function was examined in a two-sample MR design using genome-wide association study (GWAS) summary-level data. Three crucial assumptions should be hold for a valid genetic instrument: (1) it is robustly associated with exposure of interest; (2) it is not associated with potential confounders; and (3) it should exert no effect on the outcome through factors other than exposure, i.e., no horizontal pleiotropy (15). We extracted single-nucleotide polymorphism (SNP)-iron status associations from GWAS of iron status (16). SNP-lung function associations were retrieved from a meta-analysis of UK Biobank and SpiroMeta consortium (17). After data harmonization, MR effect estimates were calculated followed by multiple sensitivity analyses to test the robustness of the results. The associations of iron status with potential confounding factors were also examined.

We performed this MR study as per the Strengthening the Reporting of Observational Studies in Epidemiology-Mendelian randomization (STROBE-MR) guidelines (18). All datasets used in the present study are publicly available from each original studies, where patient consent and ethical approval had been obtained. No additional informed consent and ethical approval are required.

### Data sources for iron status

Genetic instruments associated with four serum markers of iron status, i.e., iron (μmol/l), ferritin (log_10_-transformed, ug/l), transferrin saturation (%), and transferrin (g/l) were obtained from the Genetics of Iron Status (GIS) consortium, the largest GWAS on iron status to date (16). The GIS consortium identified genetic loci associated with iron status by meta-analyzing results from 11 cohorts among 23,986 participants of European descent in its discovery phase, followed by replication in up to 24,986 participants of European descent from 8 additional cohorts (16). The mean age of participants was 46.89 ± 17.84 years, and 45% of the total participants were men. Genetic associations between SNPs and systemic iron markers were adjusted for age, principal component scores and other study specific covariates (16).

### Genetic instruments selection

SNPs showed significant associations (*P* < 5 × 10^−8^) with iron status were extracted from GIS consortium (Supplementary Table S1). A total of 5, 6, 5, and 9 SNPs were identified as genetic instruments for serum iron, ferritin, transferrin saturation, and transferrin, respectively (Supplementary Table S2). Then, SNPs with linkage disequilibrium (LD) were clumped based on 1000 Genomes LD reference panel (European population) using the TwoSampleMR package of R. All of the SNPs were in linkage equilibrium (*r*^2^ ≤ 0.01 and window size = 10 MB). One SNP, rs1799945, was missing from the FEV1 dataset without suitable proxy SNP (*r*^2^ > 0.8) was excluded. Finally, exposure and outcome SNPs were harmonized to align alleles on the forward strand. Palindromic SNPs were also omitted prior to further MR analysis.

We selected two sets of genetic instruments for iron status: (1) Conservative genetic instruments, which comprised 3 core SNPs (rs855791, rs1799945, rs1800562; Supplementary Table S1) associated with elevated serum iron, ferritin and transferrin saturation, but with decreased serum transferrin level at genome-wide significance (*P* < 5 × 10^−8^). These 3 SNPs were used for the main analyses, since systemic iron status was positively associated with serum iron, ferritin and transferrin saturation, but was negatively associated with serum transferrin level (19). (2) Liberal genetic instruments, where SNPs that were associated with at least one of the four iron biomarkers at genome-wide significance level (*P* < 5 × 10^−8^) were included (Supplementary Table S2) (16). The liberal genetic instruments contained more SNPs than conservative genetic instruments and were expected to increase the statistical power, but at the expense of increased pleiotropic bias risk.

### Data sources for lung function

3 spirometric indices of lung function were considered in current study: FEV1, FVC, and FEV1-to-FVC Ratio (FEV1/FVC). Summary-level statistics for lung function were obtained from a meta-analysis of UK Biobank (*n* = 321,047) and SpiroMeta consortium (*n* = 79,055) with up to 400,102 participants of European ancestry (17). UK Biobank is a population-based cohort study that comprises participants aged between 40 to 69 years (20). Only participants who had at least two measures of FEV1 and FVC, and complete information for age, sex, height, ever smoking status, and spirometry method used were included. Haplotype Reference Consortium was used as reference panel to impute genotypes, where age, sex, height, smoking status and genotyping array were adjusted (17). For SpiroMeta consortium, genotypes were imputed to either 1000 genomes project phase 1 panel or haplotype reference consortium panel. age, sex and height were adjusted in the model. Details of UK Biobank and SpiroMeta consortium have been described elsewhere (17, 20).

Baseline characteristics of the 400,102 participants of European ancestry included for lung function analysis are shown in the Table 1. The mean age of participants was 55.10 years, and 53.54% were men. The mean FEV1, FVC, FEV1/FVC, and PEF were 2.86, 3.74, 0.76, 406.19 L/min, respectively. Of the participants, 188,505 (47.11%) were ever smoker, and 211,597 (52.89%) were never-smokers.

**Table 1:** Baseline characteristics of participants included in the analysis.

**Characteristics**	**UK Biobank**	**SpiroMeta study**
Participants, *n*	321,047	79,055
Age, y; mean (SD)	56.44 (8.02)	49.67 (15.80)
Age at lung function measurement, y	39–72	14-99
Male (%)	178,489 (44.40)	35, 734 (45.20)
Height, cm; mean (SD)	168.57 (9.13)	168.27 (9.24)
FEV1, L; mean (SD)	2.84 (0.76)	2.98 (0.93)
FVC, L; mean (SD)	3.74 (0.96)	3.75 (1.12)
FEV1/FVC; mean (SD)	0.76 (0.06)	0.79 (0.10)
PEF, L/min; mean (SD)	406.19 (117.55)	NA
Never smokers (%)	173,658 (54.09)	37,939 (47.99)
Ever smokers (%)	147,389 (45.91)	41,116 (52.01)

FEV1, forced expired volume in 1 sec; FVC, forced vital capacity; PEF, Peak expiratory flow.

### Mendelian randomization analysis

After data harmonization, Wald ratio for individual SNP was calculated by SNP-outcome coefficient divided by SNP-exposure coefficient. Random- or fixed-effect inverse-variance weighted (IVW) method was applied to combine the Wald ratio estimates to give an overall estimate of the causal effect across all SNPs included for each iron status. This method can provide unbiased causal estimates when instrumental variables are valid and pleiotropy is absent or balanced (21). To evaluate the heterogeneity in MR analysis, Cochran's Q statistic and corresponding *P*-value for the IVW method were calculated. When significant heterogeneity was observed (*P* < 0.05), the random-effect IVW method was applied to pool the Wald ratio estimates; otherwise, the fixed-effect IVW model was used (22).

### Sensitivity analysis

MR estimates can be influenced by invalid instrument bias or potential pleiotropy. To test the validity and robustness of the MR results, we performed several sensitivity analyses: weighted median, MR-Egger regression, MR pleiotropy residual sum and outlier test (MR-PRESSO), and MR-robust adjusted profile scores (MR-RAPS) methods. Limited by the number of SNPs (*n* = 3), we can only conduct partial sensitivity analyses for the conservative instrumental variable analyses. The weighted median method can provide consistent effect estimates when more than 50% of the weight in the analysis was derived from valid genetic instruments (23). MR-Egger regression was used to check and adjust for potential directional pleiotropy. The value of intercept term significantly deviates from 0 (*P* for MR-Egger intercept < 0.05) suggests the existence of directional pleiotropy. Meanwhile, MR-Egger regression can provide pleiotropy-corrected effect estimates but with relatively low precision, particularly when the number of instrumental variables is small (24). The MR-PRESSO method was applied to detect horizontal pleiotropic outlying SNPs (25). It can also provide outlier-removed causal estimates. MR-RAPS is a method which accounts for weak instruments bias and reports pleiotropy-corrected causal effect using robust adjusted profile scores (21).

To further explore the possibility of pleiotropy, we search the online database PhenoScanner (http://www.phenoscanner.medschl.cam.ac.uk/phenoscanner) for potential secondary phenotypes associated with SNPs that were used as genetic instruments for iron status (26, 27). Two lung function-associated risk factors were considered in our analyses: body mass index (BMI) and tobacco smoking. All statistical analyses were performed using R (version 3.6.1, R Core Team, Vienna, Austria) with TwoSampleMR, MR pleiotropy residual sum and outlier, MR-robust adjusted profile scores packages. Results are reported as beta with corresponding 95% confidence interval [CI] per SD unit increase in each iron status biomarker. A 2-sided *P*-value < 0.05 was set as the threshold of statistical significance.

## Results

### Genetic instruments for iron status

Conservative and liberal genetic instruments for iron status that were used in MR analyses are shown in Supplementary Tables S1, S2, respectively. Briefly, the conservative genetic instruments contain 3 independent SNPs that have a concordant association with all of the 4 iron-related phenotypes: rs1800562, rs1799945, and rs855791 (Supplementary Table S1). For liberal genetic instruments, a total of 5, 6, 5 and 9 independent SNPs were selected for serum iron, ferritin, transferrin saturation, and transferrin, respectively (Supplementary Table S2). The total variances of iron status explained by genetic instruments were 3.4% for iron, 0.9% for (log_10_) ferritin, 6.7% for transferrin saturation, and 7.2% for transferrin (16). The F statistics for included SNPs ranged from 31 to 2947, indicating weak instrument bias was unlikely (Supplementary Table S2). We summarized the associations of genetic instruments with iron status and spirometric indices in Supplementary Tables S4, S5, respectively.

### Causal association of iron status with lung function

**Figure 2:**
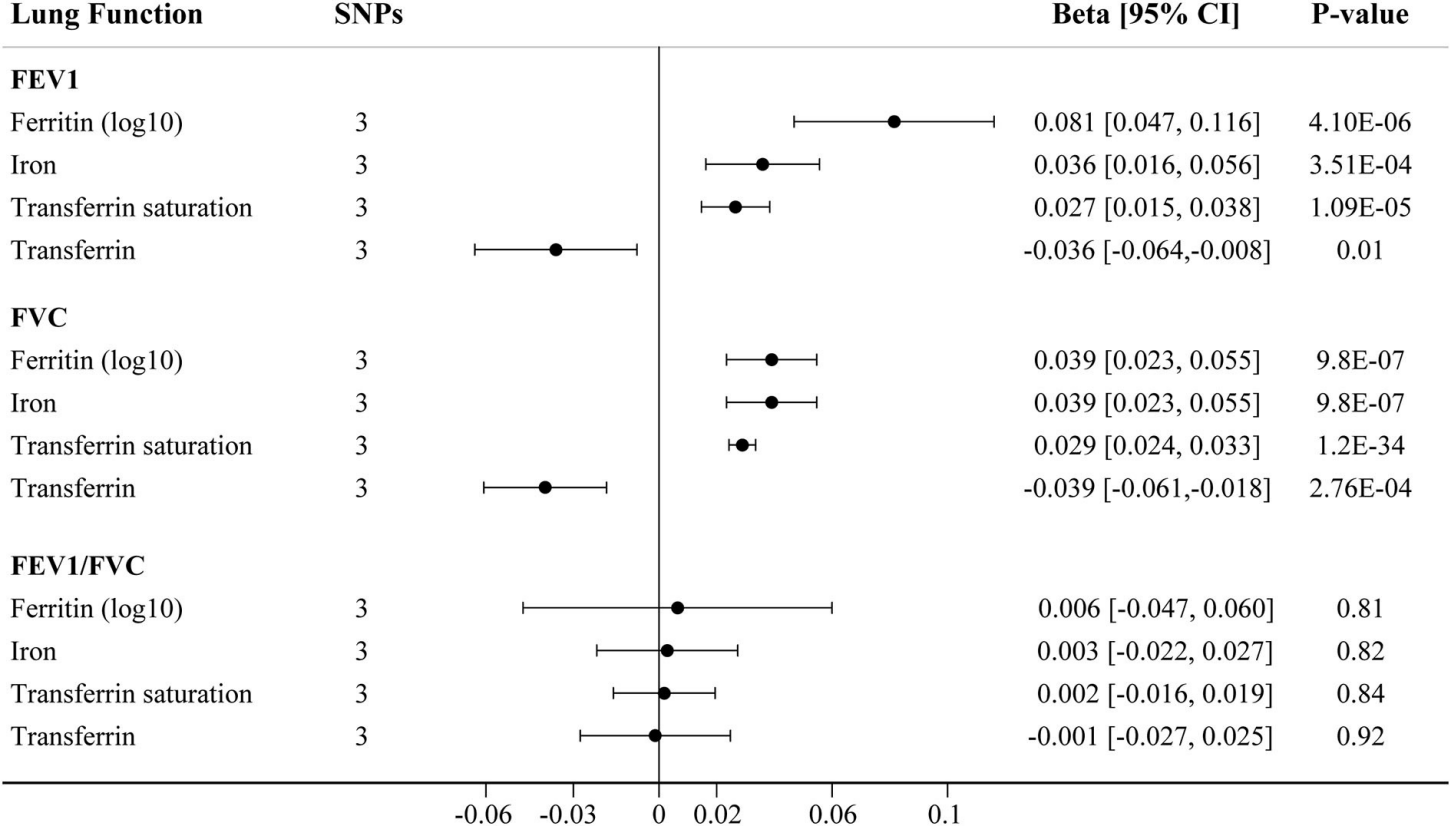
The IVW MR estimates for relationship between iron status and lung function using conservative instrumental variables associated with all four iron biomarkers. The causal effects of iron status on levels of FEV1, FVC, and FEV1/FVC ratio are estimated. Results are reported as beta with corresponding 95% CI per SD unit increase in each iron marker. IVW, inverse variance weighted; MR, Mendelian randomization; FEV1, forced expiratory volume in 1 s; FVC, forced vital capacity.

The main IVW findings between iron status and lung function (FEV1, FVC, and FEV1/FVC ratio) using conservative instrumental variables are shown in Figure 2. The results, reported as beta for spirometric indices per SD unit increase in the iron status marker, demonstrated that genetically predicted higher iron (beta = 0.036, 95% CI: 0.016 to 0.056, *P* = 3.51 × 10^−4^), log_10_-transformed ferritin (beta = 0.081, 95% CI: 0.047 to 0.116, *P* = 4.11 × 10^−6^), and transferrin saturation (beta = 0.027, 95% CI: 0.015 to 0.038, *P* = 1.09 × 10^−5^) levels were associated with increased FEV1. Genetically instrumented higher transferrin level, which reflects lower iron status, was significantly associated with decreased FEV1 (beta = −0.036, 95% CI: −0.064 to −0.008, *P* = 0.01, Figure 2). Similarly, genetical predisposition to increased levels of iron (beta = 0.039, 95%CI: 0.023 to 0.055, *P* = 9.76 × 10^−7^), log_10_-transformed ferritin (beta = 0.039, 95% CI: 0.023 to 0.055, *P* = 9.76 × 10^−7^), and transferrin saturation (beta = 0.029, 95% CI: 0.024 to 0.033, *P* = 1.23 × 10^−34^) were causally associated with higher FVC, whereas transferrin level was negatively associated with FVC (beta = −0.039, 95% CI: −0.061 to −0.018, *P* = 2.76 × 10^−4^). No significant association between iron status and FEV1/FVC ratio was observed (Figure 2).

**Figure 3:**
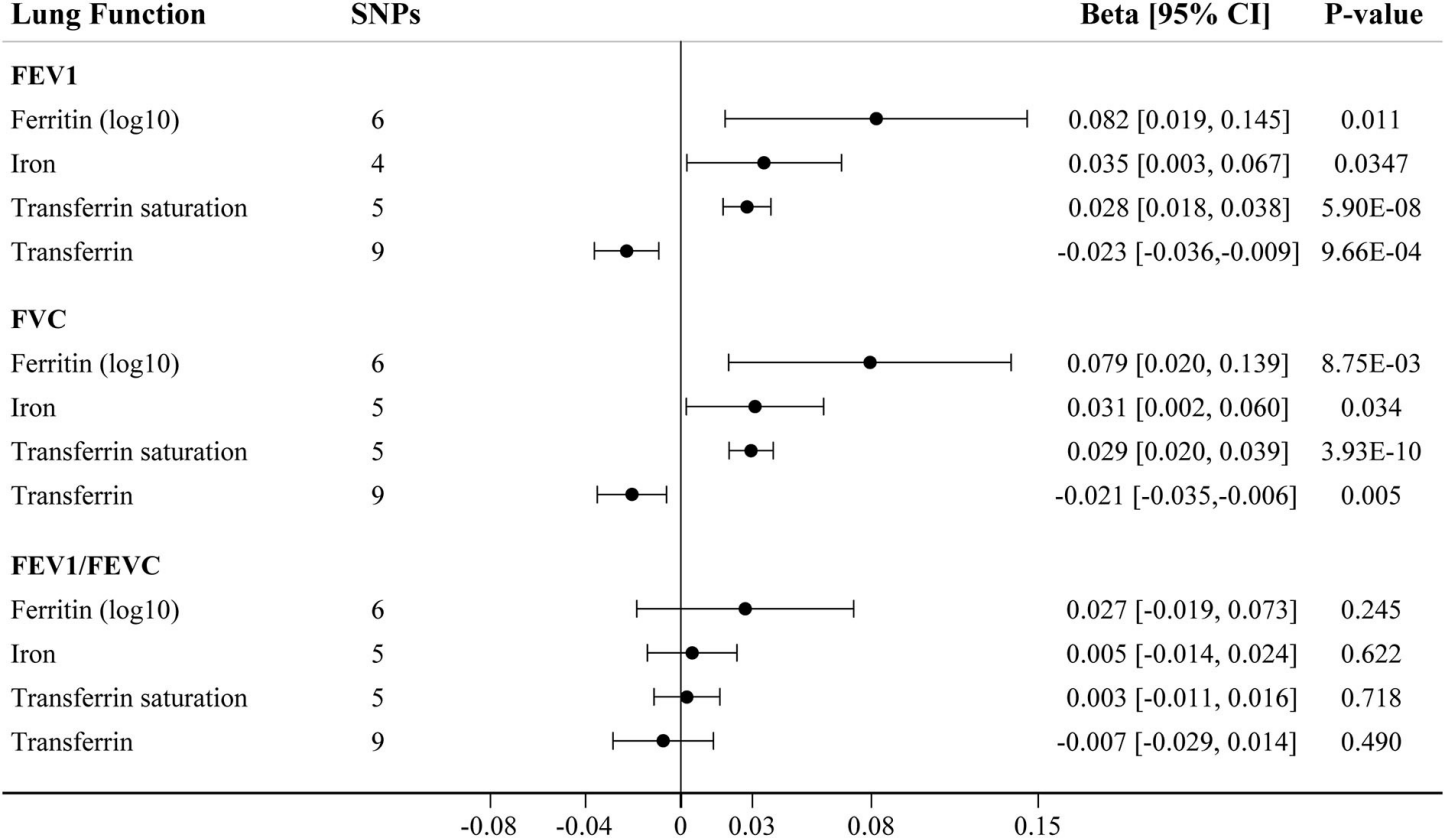
The IVW MR estimates for relationship between iron status and lung function using liberal instrumental variables associated with each iron biomarker. The causal effects of iron status on levels of FEV1, FVC, and FEV1/FVC ratio are estimated. Results are reported as beta with corresponding 95% CI per SD unit increase in each iron marker. IVW, inverse variance weighted; MR, Mendelian randomization; FEV1, forced expiratory volume in 1 s; FVC, forced vital capacity.

We further assessed the association between iron status and lung function based on liberal genetic instruments, which included more SNPs and were expected to have greater statistical power (Figure 3). Consistent results were obtained using the liberal genetic instruments, where genetically predicted iron status was positively associated with FEV1 and FVC. However, there is still no significant association between iron status and FEV1/FVC ratio using these expanded instrumental variables (Figure 3, Supplementary Table S7). None of the included SNPs exhibited any relationships with BMI or tobacco smoking (Supplementary Table S3).

### Sensitivity analysis

Using conservative instrumental variable, the causal effects of iron status on lung function were robust and consistent in all sensitivity analyses except for MR-Egger, which has wider CIs (Table 2, Supplementary Table S6). There was no substantial heterogeneity between individual SNPs in the analyses (all *P*-values for heterogeneity were >0.05). Potential pleiotropies were indicated by MR-Egger regression for the analyses of serum iron on FVC, and serum transferrin on FEV1/FVC ratio, as the intercepts were significantly departed from zero. However, the estimates from other MR methods—MR-Egger, weighted median, and MR-RAPS—were in the same direction as of those from the primary IVW analyses, providing support for the causal associations of iron status on FEV1 and FVC (Table 2). The effect estimates of each SNP on iron status and lung function outcomes are depicted in scatter plots (Supplementary Figures S1–S3). The forest plots are shown in Supplementary Figures S7–S9.

**Table 2:** Associations of genetically predicted iron status with FEV1 and FVC using the 3 SNPs associated with all 4 iron biomarkers.

**Iron status**	**Outcome**	**Method**	**No. of SNPs**	**Beta (95% CI)**	***P*-value**	***P* for heterogeneity**	***P* for intercept**
Iron	FEV1	IVW MR	3	0.036 (0.016; 0.056)	3.51E-04	0.214	0.167
		Weighted median	3	0.040 (0.021; 0.058)	4.22E-05		
		MR-Egger	3	0.073 (−0.012; 0.159)	0.342		
		MR-RAPS	3	0.037 (0.017; 0.056)	2.47E-04		
Ferritin (log_10_)	FEV1	IVW MR	3	0.081 (0.047; 0.116)	4.11E-06	0.375	0.641
		Weighted median	3	0.082 (0.044; 0.120)	2.18E-05		
		MR-Egger	3	0.072 (−0.015; 0.158)	0.353		
		MR-RAPS	3	0.081 (0.039; 0.123)	1.37E-04		
Transferrin saturation	FEV1	IVW MR	3	0.027 (0.015; 0.038)	1.09E-05	0.344	0.763
		Weighted median	3	0.029 (0.016; 0.041)	9.80E-06		
		MR-Egger	3	0.027 (−0.009; 0.063)	0.384		
		MR-RAPS	3	0.027 (0.015; 0.038)	1.05E-05		
Transferrin	FEV1	IVW MR	3	−0.036 (−0.064; −0.008)	0.012	0.062	0.183
		Weighted median	3	−0.033 (−0.051; −0.015)	3.54E-04		
		MR-Egger	3	−0.023(−0.059; 0.013)	0.429		
		MR-RAPS	3	−0.034 (−0.052; −0.016)	1.83E-04		
Iron	FVC	IVW MR	3	0.039 (0.023; 0.055)	9.76E-07	0.397	0.023
		Weighted median	3	0.039 (0.023; 0.055)	9.76E-07		
		MR-Egger	3	0.081 (0.016; 0.146)	0.248		
		MR-RAPS	3	0.039 (0.022; 0.056)	5.51E-06		
Ferritin (log_10_)	FVC	IVW MR	3	0.039 (0.023; 0.055)	9.76E-07	0.878	0.849
		Weighted median	3	0.037 (0.017; 0.056)	2.05E-04		
		MR-Egger	3	0.081 (0.016; 0.146)	0.248		
		MR-RAPS	3	0.039 (0.022; 0.056)	5.51E-06		
Transferrin saturation	FVC	IVW MR	3	0.029 (0.024; 0.033)	1.23E-34	0.854	0.790
		Weighted median	3	0.030 (0.018; 0.043)	2.78E-06		
		MR-Egger	3	0.031 (0.006; 0.056)	0.251		
		MR-RAPS	3	0.029 (0.017; 0.041)	2.48E-06		
Transferrin	FVC	IVW MR	3	−0.039(−0.061; −0.018)	2.76E-04	0.207	0.653
		Weighted median	3	−0.037(−0.055; −0.020)	2.68E-05		
		MR-Egger	3	−0.027(−0.050; −0.005)	0.255		
		MR-RAPS	3	−0.039(−0.057; −0.021)	1.40E-05		

The effect estimates were reported as beta coefficients and corresponding 95% CIs. CI, confidence interval; IVW, the inverse-variance weighted method; MR, Mendelian randomization; MR-RAPS, MR-robust adjusted profile scores; SNPs, single nucleotide polymorphisms.

In liberal instruments analyses, significant heterogeneities were observed in most analyses (Supplementary Table S7). Despite this, the heterogeneity was unlikely to influence the results, as weighted median approach provided same direction estimates compared to the main IVW MR (Table 3, Supplementary Table S7). The MR-Egger regression identified directional pleiotropy in the analyses of iron on FVC (*P* for MR-Egger intercept = 0.023), and transferrin on FEV1/FVC ratio (*P* for MR-Egger intercept = 0.012, Supplementary Table S7). However, even after correcting for pleiotropy, MR analyses still showed a causal association between serum iron and FVC (beta = 0.037, 95%CI: 0.013 to 0.060, *P* = 0.002 in MR-RAPS), and no association between transferrin and FEV1/FVC ratio (beta = −0.006, 95%CI: −0.024 to 0.012, *P* = 0.506 in MR-RAPS; beta = −0.010, 95%CI: −0.040 to 0.019, *P* = 0.522 in MR-PRESSO). The scatter plots and forest plots displaying the estimates of each SNP on lung function outcomes are shown in Supplementary Figures S4–S6, Supplementary Figures S10–S12, respectively.

**Table 3:** Associations of genetically predicted iron status with FEV1 and FVC using the separately selected SNPs associated with each iron biomarker.

**Iron status**	**Outcome**	**Method**	**No. of SNPs**	**Beta (95% CI)**	***P*-value**	***P* for heterogeneity**	***P* for intercept**
Iron	FEV1	IVW MR	4^*^	0.035 (0.003; 0.067)	0.03	0.012	0.167
		Weighted median	4^*^	0.042 (0.022; 0.061)	3.47E-05		
		MR-Egger	4^*^	0.072 (0.031; 0.112)	0.07		
		MR-RAPS	4^*^	0.040 (0.018; 0.062)	4.00E-04		
		MR-PRESSO	4^*^	0.035 (0.003; 0.067)	0.125		
Ferritin (log_10_)	FEV1	IVW MR	6	0.082 (0.019; 0.145)	0.010	2.76E-04	0.641
		Weighted median	6	0.064 (0.027; 0.101)	7.16E-04		
		MR-Egger	6	0.052 (−0.082; 0.187)	0.490		
		MR-RAPS	6	0.073 (0.028; 0.119)	0.001		
		MR-PRESSO	5^†^	0.065 (0.030; 0.100)	0.022		
Transferrin saturation	FEV1	IVW MR	5	0.028 (0.018; 0.038)	5.90E-08	0.512	0.763
		Weighted median	5	0.029 (0.016; 0.042)	7.33E-06		
		MR-Egger	5	0.025 (0.007; 0.044)	0.07		
		MR-RAPS	5	0.028 (0.016; 0.039)	1.97E-06		
		MR-PRESSO	5	0.028 (0.018; 0.038)	0.005		
Transferrin	FEV1	IVW MR	9	−0.023 (−0.036; −0.009)	9.66E-04	0.059	0.183
		Weighted median	9	−0.017(−0.029; −0.005)	0.005		
		MR-Egger	9	−0.014 (−0.031; 0.003)	0.15		
		MR-RAPS	9	−0.023 (−0.036; −0.011)	2.01E-04		
		MR-PRESSO	9	−0.023(−0.036; −0.009)	0.010		
Iron	FVC	IVW MR	5	0.031 (0.002; 0.060)	0.030	0.008	0.023
		Weighted median	5	0.034 (0.014; 0.054)	0.001		
		MR-Egger	5	0.077 (0.046; 0.107)	0.020		
		MR-RAPS	5	0.037 (0.013; 0.060)	0.002		
		MR-PRESSO	5	0.031 (0.002; 0.060)	0.101		
Ferritin (log_10_)	FVC	IVW MR	6	0.079 (0.020; 0.139)	0.008	0.001	0.849
		Weighted median	6	0.083 (0.043; 0.122)	4.20E-05		
		MR-Egger	6	0.068 (−0.060; 0.196)	0.360		
		MR-RAPS	6	0.076 (0.030; 0.122)	0.001		
		MR-PRESSO	5	0.065 (0.025; 0.105)	0.034		
Transferrin saturation	FVC	IVW MR	5	0.029 (0.020; 0.039)	3.93E-10	0.607	0.790
		Weighted median	5	0.030 (0.018; 0.043)	3.20E-06		
		MR-Egger	5	0.031 (0.014; 0.049)	0.040		
		MR-RAPS	5	0.030 (0.018; 0.041)	6.20E-07		
		MR-PRESSO	5	0.029 (0.020; 0.039)	0.003		
Transferrin	FVC	IVW MR	9	−0.021(−0.035; −0.006)	0.005	0.027	0.653
		Weighted median	9	−0.015(−0.028; −0.003)	0.020		
		MR-Egger	9	−0.024(−0.045; −0.003)	0.060		
		MR-RAPS	9	−0.024(−0.039;−0.009)	0.002		
		MR-PRESSO	9	−0.021(−0.035; −0.006)	0.024		

The effect estimates were reported as beta coefficients and corresponding 95% CIs. CI, confidence interval; IVW, the inverse-variance weighted method; MR, Mendelian randomization; MR-PRESSO, MR-Pleiotropy Residual Sum and Outlier; MR-RAPS, MR-robust adjusted profile scores; SNPs, single nucleotide polymorphisms.

* One SNP, rs1799945, was missing from FEV1 outcome dataset without suitable proxy SNP.

† Outlying SNP detected: rs651007.

## Discussion

For the first time, the causal associations of genetically instrumented systemic iron status with lung function outcomes were detailly examined using the MR design. The present MR study went beyond previous work, to provide genetic causal estimates from larger studies of lung function. Our MR analyses demonstrated consistent evidence for associations between increased iron status and higher FEV1 and FVC levels, whenever using the only 3 SNPs associated with all four iron status biomarkers (conservative genetic instruments) or additional genetic instruments associated with each iron status (liberal genetic instruments) at genome-wide significance. Specifically, we identified 27 to 81 ml increase of FEV1, and 29 to 39 ml increase of FVC per 1 SD increase in each systemic iron status. However, no association between systemic iron biomarkers and FEV1-to-FVC ratio was found. Although pleiotropy was observed in some analyses, the overall results were consistent using sensitivity analyses robust to pleiotropy.

The mechanisms underlying the association between iron status and lung function remain to be elucidated, but potentially include iron or iron-containing proteins promote lung and airway development. Iron is an important micronutrient element which has been implicated in various cellular processes such as DNA synthesis, oxygen transport, energy metabolism and mitochondrial respiration (28). Iron deficiency induced lower oxygen binding and inadequate oxygen supply could result in a growth retardation of lung during development (29). Hypoxic condition arising from iron deficiency redistributed the cardiac output to vital organs such as brain, heart and kidney, leading to reduced blood flow to the lung and restriction of lung growth (29). In animal experiments, iron chelation in lung buds from embryonic mice significantly reduced vascular branching and airway development (30). In support of these evidence, population-based investigations found that maternal iron status during pregnancy was positively associated with lung function levels in offspring at age of 10 years (31).

On the other hand, iron status may influence lung function *via* regulating immune system. Evidence from cohort studies showed that airway and lung damages caused by respiratory tract infections in early life increased the risk of FEV1 deficit in adult life (32). Iron homeostasis takes part in both innate and adaptive immunity during infections (33). In innate immunity, iron fine-tunes the function of myeloid cells through controlling the activity of enzymes and transcription factors and thus the production of antimicrobial effectors such as nitric oxide and hydroxyl radicals. Iron also plays crucial roles in adaptive immune response by regulating clonal expansion of lymphocyte subsets (34). However, iron overload or deficiency results in immune deficits may predispose organisms to respiratory infection and airway dysfunction. For instance, iron deficiency has been linked to higher airway inflammatory response and increased risk of allergic asthma (35, 36), both are involved in the pathogenesis of COPD. In contrast, iron supplement decreases the production of pro-inflammatory cytokines such as interleukin (IL)-1β, IL-9 and IL-17, and subsequently reduced airway hyper-responsiveness (37). Collectively, findings from animal studies and cell line studies provide biological evidence that iron homeostasis is beneficial for lung development, reducing airway hyper-responsiveness, and defending lung infections.

Previous observational studies reported conflicting associations between systemic iron status and lung function (8–10, 38). One study conducted in NHANES III which included participants aged above 20 years provided evidence that iron status was positively associated with FEV1 and FVC levels (39). However, another cohort study performed in 42,927 healthy Korean men suggested that higher serum ferritin, but not iron or transferrin saturation, was associated with decreased FEV1 and FVC (10). Some studies even reported null association between ferritin and lung function (8). Most studies to date linking iron status to poor lung function have co-existing risk factors for lung disease, making it difficult to rule out that lung function decline is not merely a result of confounders, rather than being a cause of iron deficiency. For example, tobacco smoking is a well-recognized risk factor for poor lung function, it can also increase the ferritin concentration in respiratory lavage (40) and serum (41), thus biasing the iron status-lung function associations in those studies. By employing two-sample MR method, we could overcome these defects of observational studies to provide strong evidence for a positive association between iron status and lung function. Moreover, the pooled MR estimates for serum iron, ferritin, transferrin saturation, and transferrin all showed that higher iron status improves FEV1 and FVC, but not FEV1/FVC ratio.

Taking advantages of the random assign of genetic variants at conception, the findings of MR studies can be treated similar to randomized controlled trails (RCTs). However, it is crucial to hold the three principal assumptions; in particular, the exclusion assumption (absence of pleiotropy), to provide unbiased causal evidence between exposure of interest and outcomes (42). To evaluate the violations of these assumptions, we searched the large-scale genetic association database PhenoScanner for potential associations of SNPs with confounders or traits that could influence lung function (26, 27). Based on prior studies, we considered BMI and tobacco smoking as potential confounding factors or sources of pleiotropy as both traits have been associated with lung function. As a result, we identified no associations of used SNPs with these phenotypes, as shown in Supplementary Table S3. However, we did not take asthma into account in our analyses, because if these SNPs have impacts on asthma, which in turn affect the lung function (vertical pleiotropy), then it still should be considered a causal factor for lung function.

There are several strengths in this MR analyses. To the best of our knowledge, this is the first MR study to examine the causal nature between systemic iron status and lung function. As genetic variants used to instrument the effect of modifying iron status were randomly allocated within participants, the frequently occurred bias in conventional epidemiological studies due to potential confounding, measurement error, and reverse causation can be largely avoided. The included genetic instruments all have strong associations with iron status (F statistics > 10) decreased weak instrument bias. Meanwhile, our analyses with large sample size (up to 400,102 participants) maximized the statistical power to detect robust causal associations. No participants overlap between exposure and outcome dataset also lowered the type 1 error rate. Moreover, multiple sensitivity analyses with different underlying assumptions (weighted median, MR-Egger, MR-PRESSO, and MR-RAPS) all provided similar and consistent estimates in our study, suggesting the credibility of our conclusion (21).

Several limitations should be considered in our work. First, the magnitude of causal estimates through genetic effect may be different from the magnitude of effect of randomized trials. This can be partly attributed to the difference in magnitude of genetic instruments and clinical interventions. In fact, MR analyses were typically designed to determine the causal association between exposure and clinical endpoints (43). Although MR study overcomes the bias from confounding, low precision in effect estimates has been deemed a tradeoff for the unconfounded association (44). Second, etiology of altered lung function is multi-factorial and many factors have been linked with lung function other than iron status. For instance, both FEV1 and FVC progressively decline with age after pulmonary maturity, and age has historically been one of the major factors in the evaluation of lung function. Gender, height and ethnic group have also been identified as contributing factors for lung function. However, as only summary-level statistics derived from general population of both genders were available in current MR study, we can hardly to conduct stratified analyses by gender, age, height and ethic group. The causal effects of iron status on lung function in these specialized populations may need further investigation. Third, all data used for genetic analyses were based on participants of European ancestry; therefore, the results are less generalizable to other populations or settings.

## Conclusion

In summary, the present MR study provides robust evidence to show that higher iron status is causally associated with higher levels of FEV1 and FVC, but has no effect on FEV1-to-FVC ratio. Our study highlights the role of systemic iron in influencing lung function among general population. Iron supplementation may be served as a therapeutic strategy for preventing the decline of lung function and associated complications in populations with iron deficiency. Further research is still needed to understand the underlying mechanisms between iron and lung function, as well as to determine the causal role of iron on lung function in specialized populations.

## Data availability statement

The original contributions presented in the study are included in the article/Supplementary material, further inquiries can be directed to the corresponding author.

## Ethics statement

Ethical review and approval was not required for the study on human participants in accordance with the local legislation and institutional requirements. The patients/participants provided their written informed consent to participate in this study.

## Author contributions

Conceptualization: ZY, CX, and FZ. Data curation and investigation: CX. Formal analysis: ZY and CF. Methodology and software: CF. Resources and writing—original draft: ZY. Supervision and writing—review and editing: FZ. All authors contributed to the article and approved the submitted version.

## References

[1] Labaki WW Rosenberg SR. Chronic obstructive pulmonary disease. Ann Intern Med. (2020) 173:ITC17–32. 10.7326/AITC202008040 32745458

[2] Global R. National incidence, prevalence, and years lived with disability for 310 diseases and injuries, 1990–2015: a systematic analysis for the global burden of disease study 2015. Lancet (London, England). (2016) 388:1545–602. 10.1016/S0140-6736(16)31678-6 27733282 PMC5055577

[3] Gershon AS Warner L Cascagnette P Victor JC To T. Lifetime risk of developing chronic obstructive pulmonary disease: a longitudinal population study. Lancet (London, England). (2011) 378:991–6. 10.1016/S0140-6736(11)60990-2 21907862

[4] Lange P Celli B Agustí A Boje Jensen G Divo M Faner R. Lung-function trajectories leading to chronic obstructive pulmonary disease. N Engl J Med. (2015) 373:111–22. 10.1056/NEJMoa1411532 26154786

[5] Martinez FD. Early-life origins of chronic obstructive pulmonary disease. N Engl J Med. (2016) 375:871–8. 10.1056/NEJMra1603287 27579637

[6] Gill D Monori G Tzoulaki I Dehghan A. Iron status and risk of stroke. Stroke. (2018) 49:2815–21. 10.1161/STROKEAHA.118.022701 30571402 PMC6257507

[7] Girelli D Marchi G Busti F Vianello A. Iron metabolism in infections: focus on COVID-19. Semin Hematol. (2021) 58:182–7. 10.1053/j.seminhematol.2021.07.001 34389110 PMC8305218

[8] Brigham EP McCormack MC Takemoto CM Matsui EC. Iron status is associated with asthma and lung function in Us women. PLoS ONE. (2015) 10:e0117545. 10.1371/journal.pone.0117545 25689633 PMC4331366

[9] Ha EK Kim JH Lee E Sung M Jee HM Baek HS. Abnormal iron status is independently associated with reduced oscillometric lung function in schoolchildren. Clin Respir J. (2021) 15:870–7. 10.1111/crj.13375 33848060

[10] Lee J Park HK Kwon M-J Ham S-Y Kim JM Lim S-Y. Decreased lung function is associated with elevated ferritin but not iron or transferrin saturation in 42,927 healthy korean men: a cross-sectional study. PLoS ONE. (2020) 15:e0231057. 10.1371/journal.pone.0231057 32240239 PMC7117746

[11] Kim T Choi H Kang J. Association of serum ferritin and lung function in tobacco-naïve postmenopausal women: analysis of population-based nationally representative data. Clin Respir J. (2020) 14:908–17. 10.1111/crj.13222 32460410

[12] Smith GD Ebrahim S. Mendelian randomization: can genetic epidemiology contribute to understanding environmental determinants of disease? Int J Epidemiol. (2003) 32:70. 10.1093/ije/dyg070 12689998

[13] Huang L Li L Luo X Huang S Hou Q Ge X. The association between serum iron status and risk of asthma: a 2-sample mendelian randomization study in descendants of Europeans. Am J Clin Nutr. (2019) 110:959–68. 10.1093/ajcn/nqz162 31380560

[14] Qin H Zeng W Lou Y. Mendelian randomization study indicates lack of causal associations between iron status and lung cancer. Medicine. (2022) 101:e29879. 10.1097/MD.0000000000029879 35866826 PMC9302260

[15] Bowden J Davey Smith G Burgess S. Mendelian randomization with invalid instruments: effect estimation and bias detection through egger regression. Int J Epidemiol. (2015) 44:512–25. 10.1093/ije/dyv080 26050253 PMC4469799

[16] Benyamin B Esko T Ried JS Radhakrishnan A Vermeulen SH Traglia M. Novel loci affecting iron homeostasis and their effects in individuals at risk for hemochromatosis. Nat Commun. (2014) 5:4926. 10.1038/ncomms5926 25352340 PMC4215164

[17] Shrine N Guyatt AL Erzurumluoglu AM Jackson VE Hobbs BD Melbourne CA. New genetic signals for lung function highlight pathways and chronic obstructive pulmonary disease associations across multiple ancestries. Nat Genet. (2019) 51:481–93. 10.1038/s41588-018-0321-7 30804560 PMC6397078

[18] Skrivankova VW Richmond RC Woolf BAR Yarmolinsky J Davies NM Swanson SA. Strengthening the reporting of observational studies in epidemiology using mendelian randomization: the strobe-Mr statement. JAMA. (2021) 326:1614–21. 10.1001/jama.2021.18236 34698778

[19] Wish JB. Assessing iron status: beyond serum ferritin and transferrin saturation. Clinical CJASN. (2006) 1(Suppl 1):S4–8. 10.2215/CJN.01490506 17699374

[20] Petersen SE Matthews PM Bamberg F Bluemke DA Francis JM Friedrich MG. Imaging in population science: cardiovascular magnetic resonance in 100,000 participants of UK Biobank—Rationale, Challenges and Approaches. J Cardiovasc Magn Reson. (2013) 15:46. 10.1186/1532-429X-15-46 23714095 PMC3668194

[21] Burgess S Davey Smith G Davies NM Dudbridge F Gill D Glymour MM. Guidelines for performing mendelian randomization investigations. Wellcome Open Research. (2019) 4:186. 10.12688/wellcomeopenres.15555.1 32760811 PMC7384151

[22] Hemani G Bowden J Davey Smith G. Evaluating the potential role of pleiotropy in mendelian randomization studies. Hum Mol Genet. (2018) 27:R195–208. 10.1093/hmg/ddy163 29771313 PMC6061876

[23] Bowden J Davey Smith G Haycock PC Burgess S. Consistent estimation in mendelian randomization with some invalid instruments using a weighted median estimator. Genet Epidemiol. (2016) 40:304–14. 10.1002/gepi.21965 27061298 PMC4849733

[24] Burgess S Bowden J Fall T Ingelsson E Thompson SG. Sensitivity analyses for robust causal inference from mendelian randomization analyses with multiple genetic variants. Epidemiology (Cambridge, Mass). (2017) 28:30–42. 10.1097/EDE.0000000000000559 27749700 PMC5133381

[25] Verbanck M Chen C-Y Neale B Do R. Detection of widespread horizontal pleiotropy in causal relationships inferred from mendelian randomization between complex traits and diseases. Nat Genet. (2018) 50:693–8. 10.1038/s41588-018-0099-7 29686387 PMC6083837

[26] Staley JR Blackshaw J Kamat MA Ellis S Surendran P Sun BB. Phenoscanner: a database of human genotype-phenotype associations. Bioinformatics (Oxford, England). (2016) 32:3207–9. 10.1093/bioinformatics/btw373 27318201 PMC5048068

[27] Gkatzionis A Burgess S. Contextualizing selection bias in mendelian randomization: how bad is it likely to be? Int J Epidemiol. (2019) 48:691–701. 10.1093/ije/dyy202 30325422 PMC6659463

[28] Muckenthaler MU Rivella S Hentze MW Galy B A. Red carpet for iron metabolism. Cell. (2017) 168:344–61. 10.1016/j.cell.2016.12.034 28129536 PMC5706455

[29] Roth-Walter F Pacios LF Bianchini R Jensen-Jarolim E. Linking iron-deficiency with allergy: role of molecular allergens and the microbiome. Metallomics. (2017) 9:1676–92. 10.1039/C7MT00241F 29120476

[30] Groenman FA Rutter M Wang J Caniggia I Tibboel D Post M. Effect of chemical stabilizers of hypoxia-inducible factors on early lung development. Am J Physiol Lung Cell Mol Physiol. (2007) 293:L557–L67. 10.1152/ajplung.00486.2006 17545484

[31] Nwaru BI Hayes H Gambling L Craig LCA Allan K Prabhu N. An exploratory study of the associations between maternal iron status in pregnancy and childhood wheeze and atopy. Br J Nutr. (2014) 112:2018–27. 10.1017/S0007114514003122 25342229

[32] Postma DS Bush A van den Berge M. Risk factors and early origins of chronic obstructive pulmonary disease. Lancet (London, England). (2015) 385:899–909. 10.1016/S0140-6736(14)60446-3 25123778

[33] Nairz M Weiss G. Iron in infection and immunity. Mol Aspects Med. (2020) 75:100864. 10.1016/j.mam.2020.100864 32461004

[34] Ganz T Nemeth E. Iron Homeostasis in host defence and inflammation. Nat Rev Immunol. (2015) 15:500–10. 10.1038/nri3863 26160612 PMC4801113

[35] Bucca C Culla B Brussino L Ricciardolo FL Cicolin A Heffler E. Effect of iron supplementation in women with chronic cough and iron deficiency. Int J Clin Pract. (2012) 66:1095–100. 10.1111/ijcp.12001 23067033

[36] Ramakrishnan K Borade A. Anemia as a risk factor for childhood asthma. Lung India. (2010) 27:51–3. 10.4103/0970-2113.63605 20616934 PMC2893424

[37] Hale LP Kant EP Greer PK Foster WM. Iron Supplementation decreases severity of allergic inflammation in murine lung. PLoS ONE. (2012) 7:e45667. 10.1371/journal.pone.0045667 23029172 PMC3447873

[38] Quezada-Pinedo HG Mensink-Bout SM Reiss IK Jaddoe VWV Vermeulen MJ Duijts L. maternal iron status during early pregnancy and school-age, lung function, asthma, and allergy: the generation R study. Pediatr Pulmonol. (2021) 56:1771–8. 10.1002/ppul.25324 33657279 PMC8251584

[39] Ghio AJ Hilborn ED. Indices of iron homeostasis correlate with airway obstruction in an nhanes Iii cohort. Int J Chron Obstruct Pulmon Dis. (2017) 12:2075–84. 10.2147/COPD.S138457 28790810 PMC5529299

[40] Ghio AJ Hilborn ED Stonehuerner JG Dailey LA Carter JD Richards JH. Particulate matter in cigarette smoke alters iron homeostasis to produce a biological effect. Am J Respir Crit Care Med. (2008) 178:1130–8. 10.1164/rccm.200802-334OC 18723436

[41] Lee CH Goag EK Lee SH Chung KS Jung JY Park MS. Association of serum ferritin levels with smoking and lung function in the korean adult population: analysis of the fourth and fifth korean national health and nutrition examination survey. Int J Chron Obstruct Pulmon Dis. (2016) 11:3001–6. 10.2147/COPD.S116982 27942209 PMC5136357

[42] Davies NM Holmes MV Davey Smith G. Reading mendelian randomisation studies: a guide, glossary, and checklist for clinicians. BMJ (Clinical research ed). (2018) 362:k601. 10.1136/bmj.k601 30002074 PMC6041728

[43] Ference BA Julius S Mahajan N Levy PD Williams KA Flack JM. Clinical effect of naturally random allocation to lower systolic blood pressure beginning before the development of hypertension. Hypertension (Dallas, Tex: 1979). (2014). 63:1182–8. 10.1161/HYPERTENSIONAHA.113.02734 24591335

[44] SG BST. Mendelian Randomization: Methods for Using Genetic Variants in Causal Estimation.. London, UK: Chapman and Hall/CRC (2015).

