# Supplementary material for: Causal effect of iron status on lung function: A Mendelian randomization study
Source: Front Nutr. 2022 Dec 15;9:1025212. doi: 10.3389/fnut.2022.1025212 (PMC9798299; doi:10.3389/fnut.2022.1025212)
Supplement: Supplementary file 1 [file Table_1.docx]

**Causal Effect of Iron Status on Lung Function: A Mendelian Randomization Study**

Zhimin Yu, Chengkai Xu, Chenggang Fang, Fangfang Zhang

Online Supplement

**Supplementary Table S1**. Detailed information of 3 SNPs associated with all 4 iron status biomarkers.

**Supplementary Table S2**. Detailed information of LD-independent SNPs (after clumping process) for each iron status biomarker.

**Supplementary Table S3**. Association of the SNPs used as instruments (P<5x10^-8^) with confounders or lung function risk factors in PhenoScanner (accessed on 2022/07/24).

**Supplementary Table S4**. Effect estimates of the associations between conservative genetic instruments for iron status and lung function.

**Supplementary Table S5**. Effect estimates of the associations between liberal genetic instruments for iron status and lung function.

**Supplementary Table S6**. MR estimates from different methods of assessing the causal effects of iron status on lung function based on conservative genetic instruments.

**Supplementary Table S7.** MR estimates from different methods of assessing the causal effects of iron status on lung function based on liberal genetic instruments.

**Supplementary Table S1.** Detailed information of 3 SNPs associated with all 4 iron status biomarkers.

| **SNP** | **CHR** | **BP** | **Iron status** | **F statistics** | **EA** | **OA** | **EAF** | **Beta** | **SE** | **P-value** |
| --- | --- | --- | --- | --- | --- | --- | --- | --- | --- | --- |
| rs1800562 | 6 | 26,093,141 | Iron | 420 | A | G | 0.067 | 0.328 | 0.016 | 2.72 E-97 |
| rs1799945 | 6 | 26,091,179 | Iron | 357 | C | G | 0.85 | -0.189 | 0.01 | 1.10E-81 |
| rs855791 | 22 | 37,462,936 | Iron | 669 | A | G | 0.446 | -0.181 | 0.007 | 1.32E-139 |
| rs1800562 | 6 | 26,093,141 | Ferritin (log_10_) | 163 | A | G | 0.067 | 0.204 | 0.016 | 1.54E-38 |
| rs1799945 | 6 | 26,091,179 | Ferritin (log_10_) | 42 | C | G | 0.85 | -0.065 | 0.01 | 1.71E-10 |
| rs855791 | 22 | 37,462,936 | Ferritin (log_10_) | 62 | A | G | 0.446 | -0.055 | 0.007 | 1.38E-14 |
| rs1800562 | 6 | 26,093,141 | Transferrin saturation | 1301 | A | G | 0.067 | 0.577 | 0.016 | 2.19E-270 |
| rs1799945 | 6 | 26,091,179 | Transferrin saturation | 534 | C | G | 0.85 | -0.231 | 0.01 | 5.13E-109 |
| rs855791 | 22 | 37,462,936 | Transferrin saturation | 564 | A | G | 0.446 | -0.19 | 0.008 | 6.41E-137 |
| rs1800562 | 6 | 26,093,141 | Transferrin | 896 | A | G | 0.067 | -0.479 | 0.016 | 8.90E-196 |
| rs1799945 | 6 | 26,091,179 | Transferrin | 130 | C | G | 0.85 | 0.114 | 0.01 | 9.36E-30 |
| rs855791 | 22 | 37,462,936 | Transferrin | 40 | A | G | 0.446 | 0.044 | 0.007 | 1.98E-09 |

**Supplementary Table S2.** Detailed information of LD-independent SNPs (after clumping process) for each iron status biomarker.

| **SNP** | **CHR** | **BP** | **Iron status** | **F statistics** | **EA** | **OA** | **EAF** | **Beta** | **SE** | **P-value** |
| --- | --- | --- | --- | --- | --- | --- | --- | --- | --- | --- |
| rs8177240 | 3 | 133,477,701 | Iron | 89 | T | G | 0.669 | -0.066 | 0.007 | 6.65E-20 |
| rs1800562 | 6 | 26,093,141 | Iron | 420 | A | G | 0.067 | 0.328 | 0.016 | 2.72 E-97 |
| rs1799945 | 6 | 26,091,179 | Iron | 357 | C | G | 0.85 | -0.189 | 0.010 | 1.10E-81 |
| rs7385804 | 7 | 100,235,970 | Iron | 84 | A | C | 0.621 | 0.064 | 0.007 | 1.36E-18 |
| rs855791 | 22 | 37,462,936 | Iron | 669 | A | G | 0.446 | -0.181 | 0.007 | 1.32E-139 |
| rs744653 | 2 | 190,378,750 | Transferrin | 46 | T | C | 0.854 | 0.068 | 0.010 | 1.35E-11 |
| rs8177240 | 3 | 133,477,701 | Transferrin | 2947 | T | G | 0.669 | -0.38 | 0.007 | 3.29E-615 |
| rs9990333 | 3 | 195,827,205 | Transferrin | 53 | T | C | 0.46 | -0.051 | 0.007 | 1.95E-13 |
| rs1800562 | 6 | 26,093,141 | Transferrin | 896 | A | G | 0.067 | -0.479 | 0.016 | 8.90E-196 |
| rs1799945 | 6 | 26,091,179 | Transferrin | 130 | C | G | 0.85 | 0.114 | 0.010 | 9.36E-30 |
| rs4921915 | 8 | 18,272,466 | Transferrin | 77 | A | G | 0.782 | 0.079 | 0.009 | 7.05E-19 |
| rs6486121 | 11 | 13,355,770 | Transferrin | 43 | T | C | 0.631 | -0.046 | 0.007 | 3.89E-10 |
| rs174577 | 11 | 61,604,814 | Transferrin | 78 | A | C | 0.33 | 0.062 | 0.007 | 2.28E-17 |
| rs855791 | 22 | 37,462,936 | Transferrin | 40 | A | G | 0.446 | 0.044 | 0.007 | 1.98E-09 |
| rs8177240 | 3 | 133,477,701 | Transferrin saturation | 156 | T | G | 0.669 | 0.1 | 0.008 | 7.24E-38 |
| rs1800562 | 6 | 26,093,141 | Transferrin saturation | 1301 | A | G | 0.067 | 0.577 | 0.016 | 2.19E-270 |
| rs1799945 | 6 | 26,091,179 | Transferrin saturation | 534 | C | G | 0.85 | -0.231 | 0.010 | 5.13E-109 |
| rs7385804 | 7 | 100,235,970 | Transferrin saturation | 46 | A | C | 0.621 | 0.054 | 0.008 | 6.07E-12 |
| rs855791 | 22 | 37,462,936 | Transferrin saturation | 564 | A | G | 0.446 | -0.19 | 0.008 | 6.41E-137 |
| rs744653 | 2 | 190,378,750 | Ferritin (log_10_) | 79 | T | C | 0.854 | -0.089 | 0.010 | 8.37E-19 |
| rs1800562 | 6 | 26,093,141 | Ferritin (log_10_) | 163 | A | G | 0.067 | 0.204 | 0.016 | 1.54E-38 |
| rs1799945 | 6 | 26,091,179 | Ferritin (log_10_) | 42 | C | G | 0.85 | -0.065 | 0.010 | 1.71E-10 |
| rs651007 | 9 | 136,153,875 | Ferritin (log_10_) | 31 | T | C | 0.202 | -0.05 | 0.009 | 1.31E-08 |
| rs411988 | 17 | 56,709,034 | Ferritin (log_10_) | 40 | A | G | 0.564 | -0.044 | 0.007 | 1.59E-10 |
| rs855791 | 22 | 37,462,936 | Ferritin (log_10_) | 62 | A | G | 0.446 | -0.055 | 0.007 | 1.38E-14 |

**Supplementary Table S3.** Association of the SNPs used as instruments (P<5x10-8) with confounders or lung function risk factors in PhenoScanner (accessed on 2022/07/24).

| SNP | Confounders/ risk factors |
| --- | --- |
| rs8177240 | - |
| rs1800562 | - |
| rs1799945 | - |
| rs7385804 | - |
| rs855791 | - |
| rs744653 | - |
| rs9990333 | - |
| rs4921915 | - |
| rs6486121 | - |
| rs174577 | - |
| rs651007 | - |
| rs411988 | - |

Tobacco smoking and BMI were considered as relevant confounders or risk factors for pulmonary function.

**Supplementary Table S4.** Effect estimates of the associations between conservative genetic instruments for iron status and lung function.

| **SNP** | **Iron status** | **EA** | **OA** | **EAF** | **Exposure** | | | **Lung Function** | | | | | | | | |
| --- | --- | --- | --- | --- | --- | --- | --- | --- | --- | --- | --- | --- | --- | --- | --- | --- |
|  |  |  |  |  |  |  |  | **FEV1** | | | **FVC** | | | **FEV1/FVC** | | |
|  |  |  |  |  | **Beta** | **SE** | **P-value** | **Beta** | **SE** | **P-value** | **Beta** | **SE** | **P-value** | **Beta** | **SE** | **P-value** |
| rs1799945 | iron | C | G | 0.85 | -0.189 | 0.01 | 1.10E-81 | -0.002 | 0.003 | 0.4894 | -0.005 | 0.003 | 0.123 | 0.005 | 0.003 | 0.112 |
| rs1800562 | iron | A | G | 0.06 | 0.328 | 0.016 | 1.08E-93 | 0.016 | 0.004 | 2.18E-04 | 0.017 | 0.004 | 8.40E-05 | 0.002 | 0.004 | 0.653 |
| rs855791 | Iron | A | G | 0.44 | -0.181 | 0.007 | 1.32E-139 | -0.006 | 0.002 | 0.003 | -0.005 | 0.002 | 0.012 | -0.003 | 0.002 | 0.181 |
| rs1799945 | Ferritin (log_10_) | C | G | 0.85 | -0.065 | 0.010 | 1.71E-10 | -0.002 | 0.003 | 0.489 | -0.005 | 0.003 | 0.123 | 0.005 | 0.003 | 0.112 |
| rs1800562 | Ferritin (log_10_) | A | G | 0.06 | 0.204 | 0.016 | 1.54E-38 | 0.016 | 0.004 | 2.18E-04 | 0.017 | 0.004 | 8.40E-05 | 0.002 | 0.004 | 0.653 |
| rs855791 | Ferritin (log_10_) | A | G | 0.44 | -0.055 | 0.007 | 1.38E-14 | -0.006 | 0.002 | 0.003 | -0.005 | 0.002 | 0.012 | -0.003 | 0.002 | 0.181 |
| rs1799945 | Transferrin saturation | C | G | 0.85 | -0.231 | 0.010 | 5.13E-109 | -0.002 | 0.003 | 0.489 | -0.005 | 0.003 | 0.123 | 0.005 | 0.003 | 0.112 |
| rs1800562 | Transferrin saturation | A | G | 0.06 | 0.577 | 0.016 | 1.00E-200 | 0.016 | 0.004 | 2.18E-04 | 0.0174 | 0.004 | 8.40E-05 | 0.002 | 0.004 | 0.653 |
| rs855791 | Transferrin saturation | A | G | 0.44 | -0.190 | 0.008 | 6.41E-137 | -0.006 | 0.002 | 0.003 | -0.005 | 0.002 | 0.012 | -0.003 | 0.002 | 0.181 |
| rs1799945 | Transferrin | C | G | 0.85 | 0.114 | 0.01 | 9.36E-30 | -0.002 | 0.003 | 0.489 | -0.005 | 0.003 | 0.123 | 0.005 | 0.003 | 0.112 |
| rs1800562 | Transferrin | A | G | 0.06 | -0.479 | 0.016 | 8.90E-196 | 0.016 | 0.004 | 2.18E-04 | 0.017 | 0.004 | 8.40E-05 | 0.002 | 0.004 | 0.653 |
| rs855791 | Transferrin | A | G | 0.44 | 0.044 | 0.007 | 1.98E-09 | -0.006 | 0.002 | 0.003 | -0.005 | 0.002 | 0.012 | -0.003 | 0.002 | 0.181 |

**Supplementary Table S5.** Effect estimates of the associations between liberal genetic instruments for iron status and lung function.

| **SNP** | **Iron status** | **EA** | **OA** | **EAF** | **Exposure** | | | **Lung Function** | | | | | | | | |
| --- | --- | --- | --- | --- | --- | --- | --- | --- | --- | --- | --- | --- | --- | --- | --- | --- |
|  |  |  |  |  |  |  |  | **FEV1** | | | **FVC** | | | **FEV1/FVC** | | |
|  |  |  |  |  | **Beta** | **SE** | **P-value** | **Beta** | **SE** | **P-value** | **Beta** | **SE** | **P-value** | **Beta** | **SE** | **P-value** |
| rs1799945 | Iron | C | G | 0.85 | -0.189 | 0.010 | 1.10E-81 | NA | NA | NA | -0.005 | 0.0033 | 0.123 | 0.005 | 0.003 | 0.112 |
| rs1800562 | Iron | A | G | 0.06 | 0.328 | 0.016 | 1.08E-93 | 0.016 | 0.004 | 2.18E-04 | 0.0174 | 0.0044 | 8.40E-05 | 0.002 | 0.004 | 0.653 |
| rs7385804 | Iron | A | C | 0.62 | 0.064 | 0.007 | 1.36E-18 | 8.00E-04 | 0.002 | 0.748 | -0.0011 | 0.0024 | 0.64 | 0.003 | 0.002 | 0.213 |
| rs8177240 | Iron | T | G | 0.66 | -0.066 | 0.007 | 6.65E-20 | 0.005 | 0.002 | 0.030 | 0.0056 | 0.0025 | 0.023 | -1.00E-04 | 0.002 | 0.983 |
| rs855791 | Iron | A | G | 0.44 | -0.181 | 0.007 | 1.32E-139 | -0.006 | 0.002 | 0.003 | -0.0059 | 0.0024 | 0.0124 | -0.003 | 0.002 | 0.181 |
| rs1799945 | Ferritin | C | G | 0.85 | -0.065 | 0.01 | 1.71E-10 | -0.002 | 0.003 | 0.489 | -0.005 | 0.0033 | 0.123 | 0.005 | 0.003 | 0.112 |
| rs1800562 | Ferritin | A | G | 0.06 | 0.204 | 0.016 | 1.54E-38 | 0.016 | 0.004 | 2.17E-04 | 0.0174 | 0.0044 | 8.40E-05 | 0.002 | 0.004 | 0.653 |
| rs411988 | Ferritin | A | G | 0.56 | -0.044 | 0.007 | 1.59E-10 | -0.001 | 0.002 | 0.592 | 2.00E-04 | 0.0023 | 0.923 | -0.004 | 0.002 | 0.093 |
| rs651007 | Ferritin | T | C | 0.20 | -0.050 | 0.009 | 1.31E-08 | -0.016 | 0.002 | 2.52E-08 | -0.0142 | 0.002 | 8.96E-07 | -0.007 | 0.002 | 0.010 |
| rs744653 | Ferritin | T | C | 0.85 | -0.089 | 0.010 | 8.37E-19 | -8.00E-04 | 0.003 | 0.821 | 3.00E-04 | 0.003 | 0.939 | -0.002 | 0.003 | 0.423 |
| rs855791 | Ferritin | A | G | 0.44 | -0.055 | 0.007 | 1.38E-14 | -0.0068 | 0.002 | 0.003 | -0.005 | 0.002 | 0.012 | -0.003 | 0.002 | 0.181 |
| rs1799945 | Transferrin saturation | C | G | 0.85 | -0.231 | 0.010 | 5.13E-109 | -0.002 | 0.003 | 0.489 | -0.005 | 0.003 | 0.123 | 0.005 | 0.003 | 0.112 |
| rs1800562 | Transferrin saturation | A | G | 0.06 | 0.577 | 0.016 | 1.00E-200 | 0.016 | 0.004 | 2.18E-04 | 0.017 | 0.004 | 8.40E-05 | 0.002 | 0.004 | 0.653 |
| rs7385804 | Transferrin saturation | A | C | 0.62 | 0.054 | 0.008 | 6.07E-12 | 8.00E-04 | 0.002 | 0.748 | -0.001 | 0.002 | 0.64 | 0.003 | 0.002 | 0.213 |
| rs8177240 | Transferrin saturation | T | G | 0.66 | 0.100 | 0.008 | 7.24E-38 | 0.005 | 0.002 | 0.030 | 0.005 | 0.002 | 0.023 | -1.00E-04 | 0.002 | 0.983 |
| rs855791 | Transferrin saturation | A | G | 0.44 | -0.190 | 0.008 | 6.41E-137 | -0.006 | 0.002 | 0.003 | -0.005 | 0.002 | 0.012 | -0.003 | 0.002 | 0.181 |
| rs174577 | Transferrin | A | C | 0.33 | 0.062 | 0.007 | 2.28E-17 | 8.00E-04 | 0.002 | 0.735 | 0.004 | 0.002 | 0.060 | -0.007 | 0.002 | 0.004 |
| rs1799945 | Transferrin | C | G | 0.85 | 0.114 | 0.010 | 9.36E-30 | -0.002 | 0.003 | 0.489 | -0.005 | 0.003 | 0.123 | 0.005 | 0.003 | 0.112 |
| rs1800562 | Transferrin | A | G | 0.06 | -0.479 | 0.016 | 8.90E-196 | 0.016 | 0.004 | 2.18E-04 | 0.0174 | 0.004 | 8.40E-05 | 0.002 | 0.004 | 0.653 |
| rs4921915 | Transferrin | A | G | 0.78 | 0.079 | 0.009 | 7.05E-19 | -0.002 | 0.002 | 0.480 | 0.003 | 0.002 | 0.283 | -0.008 | 0.0028 | 0.005 |
| rs6486121 | Transferrin | T | C | 0.63 | -0.046 | 0.007 | 3.89E-10 | 0.005 | 0.002 | 0.020 | 9.00E-04 | 0.002 | 0.699 | 0.008 | 0.0025 | 4.72E-04 |
| rs744653 | Transferrin | T | C | 0.85 | 0.068 | 0.010 | 1.35E-11 | -8.00E-04 | 0.003 | 0.821 | 3.00E-04 | 0.003 | 0.939 | -0.002 | 0.003 | 0.423 |
| rs8177240 | Transferrin | T | G | 0.66 | -0.380 | 0.007 | 1.00E-200 | 0.005 | 0.002 | 0.030 | 0.005 | 0.002 | 0.023 | -1.00E-04 | 0.002 | 0.983 |
| rs855791 | Transferrin | A | G | 0.44 | 0.044 | 0.007 | 1.98E-09 | -0.006 | 0.002 | 0.003 | -0.005 | 0.002 | 0.012 | -0.003 | 0.002 | 0.181 |
| rs9990333 | Transferrin | T | C | 0.46 | -0.051 | 0.007 | 1.95E-13 | 0.003 | 0.002 | 0.152 | 8.00E-04 | 0.002 | 0.746 | 0.005 | 0.002 | 0.033 |

**Supplementary Table S6.** MR estimates from different methods of assessing the causal effects of iron status on lung function based on conservative genetic instruments.

|  | **IVW** | | | |  | **Weighted Median** | |  | **MR.RAPS** | |  | **MR-Egger** | | | |
| --- | --- | --- | --- | --- | --- | --- | --- | --- | --- | --- | --- | --- | --- | --- | --- |
| **Lung function** | **Beta**  **(95% CI)** | **P-value** | **Cochran**  **Q statistics** | **P-value** |  | **Beta**  **(95% CI)** | **P-value** |  | **Beta**  **(95% CI)** | **P-value** |  | **Beta**  **(95% CI)** | **P-value** | **Intercept**  **(SE)** | **P-value** |
| **FEV1** |  |  |  |  |  |  |  |  |  |  |  |  |  |  |  |
| Iron | 0.036  (0.016; 0.056) | **3.51E-04** | 3.08 | 0.214 |  | 0.040 (0.021;0.058) | **4.22E-05** |  | 0.037 (0.017;0.056) | **2.47E-04** |  | 0.073  (-0.012; 0.159) | 0.342 | -0.006 (0.003) | 0.167 |
| Ferritin | 0.081  (0.047; 0.116) | **4.11E-06** | 1.96 | 0.375 |  | 0.082  (0.044; 0.120) | **2.18E-05** |  | 0.081  (0.039; 0.123) | **1.37E-04** |  | 0.072  (-0.015; 0.158) | 0.353 | 0.002 (0.005) | 0.641 |
| Transferrin saturation | 0.027  (0.015; 0.038) | **1.09E-05** | 2.13 | 0.344 |  | 0.029  (0.016; 0.041) | **9.80E-06** |  | 0.027  (0.015; 0.038) | **1.05E-05** |  | 0.027  (-0.009; 0.063) | 0.384 | 0.000 (0.002) | 0.763 |
| Transferrin | -0.036  (-0.064;-0.008) | **0.012** | 5.55 | 0.062 |  | -0.033  (-0.051; -0.015) | **3.54E-04** |  | -0.034  (-0.052;-0.016) | **1.83E-04** |  | -0.023  (-0.059; 0.013) | 0.429 | -0.002 (0.001) | 0.183 |
| **FVC** |  |  |  |  |  |  |  |  |  |  |  |  |  |  |  |
| Iron | 0.039  (0.023; 0.055) | **9.76E-07** | 1.84 | 0.397 |  | 0.037  (0.017; 0.056) | **2.05E-04** |  | 0.039  (0.022;0.056) | **5.51E-06** |  | 0.081  (0.016; 0.146) | 0.248 | -0.008 (0.001) | **0.023** |
| Ferritin | 0.039  (0.023; 0.055) | **9.76E-07** | 0.25 | 0.878 |  | 0.037  (0.017; 0.056) | **2.05E-04** |  | 0.039  (0.022;0.056) | **5.51E-06** |  | 0.081  (0.016; 0.146) | 0.248 | 0.001 (0.005) | 0.849 |
| Transferrin saturation | 0.029  (0.024; 0.033) | **1.23E-34** | 0.31 | 0.854 |  | 0.030  (0.018; 0.043) | **2.78E-06** |  | 0.029  (0.017;0.041) | **2.48E-06** |  | 0.031  (0.006; 0.056) | 0.251 | -0.000 (0.001) | 0.790 |
| Transferrin | -0.039  (-0.061;-0.018) | **2.76E-04** | 3.14 | 0.207 |  | -0.037  (-0.055; -0.020) | **2.68E-05** |  | -0.039  (-0.057;-0.021) | **1.40E-05** |  | -0.027  (-0.050; -0.005) | 0.255 | 0.000 (0.001) | 0.653 |
| **FEV1/FVC** |  |  |  |  |  |  |  |  |  |  |  |  |  |  |  |
| Iron | 0.003  (-0.022;0.027) | 0.817 | 4.43 | 0.108 |  | 0.009  (-0.011;0.029) | 0.393 |  | 0.003  (-0.017;0.023) | 0.778 |  | 0.007  (-0.132;0.147) | 0.934 | 0.001 (0.003) | 0.645 |
| Ferritin | 0.006  (-0.047;0.060) | 0.814 | 4.43 | 0.109 |  | 0.013  (-0.027;0.052) | 0.532 |  | 0.006  (-0.035;0.048) | 0.765 |  | 0.006  (-0.132;0.145) | 0.944 | 0.004 (0.003) | 0.308 |
| Transferrin saturation | 0.002  (-0.016;0.019) | 0.839 | 4.46 | 0.107 |  | 0.004  (-0.010;0.018) | 0.544 |  | 0.002  (-0.012;0.016) | 0.805 |  | 0.001  (-0.053;0.055) | 0.976 | 0.001 (0.002) | 0.610 |
| Transferrin | -0.001  (-0.027;0.025) | 0.924 | 4.53 | 0.103 |  | -0.001  (-0.019;0.018) | 0.937 |  | -0.001  (-0.021;0.018) | 0.886 |  | 0.001  (-0.048;0.049) | 0.978 | -0.005 (0.001) | **0.012** |

P-values below the significance threshold of 0.05 are displayed in bold.

Abbreviations: CI = confidence interval; FEV1 = forced expiratory volume in 1 second; FVC = forced vital capacity; IVW =invariance weighted; MR.RAPS = MR-robust adjusted profile scores.

**Supplementary Table S7.** MR estimates from different methods of assessing the causal effects of iron status on lung function based on liberal genetic instruments.

|  |  | **IVW** | | | |  | **MR.RAPS** | |  | **Weighted Median** | |  | **MR-Egger** | | |  |  | **MR-PRESSO** | | |
| --- | --- | --- | --- | --- | --- | --- | --- | --- | --- | --- | --- | --- | --- | --- | --- | --- | --- | --- | --- | --- |
| **Lung function** | **SNPs** | **Beta**  **(95% CI)** | **P-value** | **Cochran**  **Q statistics** | ***P*_heterogeneity_** |  | **Beta**  **(95% CI)** | **P-value** |  | **Beta**  **(95% CI)** | **P-value** |  | **Beta**  **(95% CI)** | **P-value** | **Intercept**  **(SE)** | **P-value** |  | **SNPs** | **Beta**  **(95% CI)** | **P-value** |
| **FEV1** |  |  |  |  |  |  |  |  |  |  |  |  |  |  |  |  |  |  |  |  |
| Iron | 4^1^ | 0.035  (0.003; 0.067) | **0.034** | 10.81 | **0.012** |  | 0.040  (0.018;0.062) | **4.00E-04** |  | 0.042  (0.022; 0.061) | **3.47E-05** |  | 0.072  (0.031; 0.112) | 0.073 | -0.007 (0.003) | 0.167 |  | 4^1^ | 0.035  (0.003;0.067) | 0.125 |
| Ferritin | 6 | 0.082  (0.019; 0.145) | **0.011** | 23.46 | **2.76E-04** |  | 0.073  (0.028;0.119) | **0.001** |  | 0.064  (0.027; 0.101) | **7.16E-04** |  | 0.052  (-0.082; 0.187) | 0.487 | 0.003 (0.005) | 0.641 |  | 5* | 0.065  (0.030;0.100) | **0.022** |
| Transferrin saturation | 5 | 0.028  (0.018; 0.038) | **5.90E-08** | 3.27 | 0.512 |  | 0.028  (0.016;0.039) | **1.97E-06** |  | 0.029  (0.016; 0.042) | **7.33E-06** |  | 0.025  (0.007; 0.044) | 0.072 | 0.001 (0.002) | 0.763 |  | 5 | 0.028  (0.018;0.038) | **0.005** |
| Transferrin | 9 | -0.023  (-0.036; -0.009) | **9.66E-04** | 14.98 | 0.059 |  | -0.023  (-0.036;-0.011) | **2.01E-04** |  | -0.017  (-0.029;-0.005) | **0.005** |  | -0.014  (-0.031; 0.003) | 0.151 | -0.002 (0.002) | 0.183 |  | 9 | -0.023  (-0.036;-0.009) | **0.010** |
| **FVC** |  |  |  |  |  |  |  |  |  |  |  |  |  |  |  |  |  |  |  |  |
| Iron | 5 | 0.031  (0.002;0.060) | **0.034** | 13.78 | **0.008** |  | 0.037  (0.013;0.060) | **0.002** |  | 0.034  (0.014; 0.054) | **0.001** |  | 0.077  (0.046; 0.107) | **0.015** | -0.008 (0.002) | **0.023** |  | 5 | 0.031  (0.002;0.06) | 0.101 |
| Ferritin | 6 | 0.079  (0.02; 0.139) | **0.008** | 20.20 | **0.001** |  | 0.076  (0.030;0.122) | **0.001** |  | 0.083  (0.043; 0.122) | **4.20E-05** |  | 0.068  (-0.06; 0.196) | 0.357 | 0.001 (0.005) | 0.849 |  | 5 | 0.065  (0.025;0.105) | **0.034** |
| Transferrin saturation | 5 | 0.029  (0.02; 0.039) | **3.93E-10** | 2.70 | 0.607 |  | 0.03  (0.018;0.041) | **6.20E-07** |  | 0.030  (0.018; 0.043) | **3.20E-06** |  | 0.031  (0.014; 0.049) | 4.08E-02 | -0.001 (0.002) | 0.790 |  | 5 | 0.029  (0.02;0.039) | **0.003** |
| Transferrin | 9 | -0.021  (-0.035;-0.006) | **0.005** | 17.21 | **0.027** |  | -0.024  (-0.039;-0.009) | **0.002** |  | -0.015  (-0.028;-0.003) | **0.017** |  | -0.024  (-0.045;-0.003) | 0.058 | 0.001 (0.002) | 0.653 |  | 9 | -0.021  (-0.035;-0.006) | **0.024** |
| **FEV1/FVC** |  |  |  |  |  |  |  |  |  |  |  |  |  |  |  |  |  |  |  |  |
| Iron | 5 | 0.005  (-0.014;0.024) | 0.822 | 5.74 | 0.218 |  | 0.007  (-0.01;0.023) | 0.420 |  | 0.009  (-0.011;0.028) | 0.392 |  | -0.004  (-0.045;0.036) | 0.844 | 0.002 (0.003) | 0.645 |  | 5 | 0.005  (-0.014;0.024) | 0.648 |
| Ferritin | 6 | 0.027  (-0.019;0.073) | 0.245 | 11.57 | **0.041** |  | 0.022  (-0.019;0.063) | 0.295 |  | 0.021  (-0.017;0.058) | 0.277 |  | -0.017  (-0.103;0.069) | 0.717 | 0.004 (0.003) | 0.308 |  | 6 | 0.027  (-0.019;0.073) | 0.297 |
| Transferrin saturation | 5 | 0.003  (-0.011;0.016) | 0.718 | 5.90 | 0.206 |  | 0.003  (-0.009;0.016) | 0.603 |  | 0.004  (-0.01;0.017) | 0.591 |  | -0.003  (-0.027;0.021) | 0.826 | 0.002 (0.003) | 0.610 |  | 5 | 0.005  (-0.008;0.018) | 0.737 |
| Transferrin | 9 | -0.007  (-0.029;0.014) | 0.490 | 35.68 | 2.00E-05 |  | -0.006  (-0.024;0.012) | 0.506 |  | -0.001  (-0.012;0.009) | 0.792 |  | 0.014  (-0.005;0.034) | 0.180 | -0.006 (0.002) | **0.012** |  | 6* | -0.010  (-0.04;0.019) | 0.522 |

^1^ rs1799945 was missing from outcome dataset without suitable proxy SNP.

* One or more SNPs excluded.

P-values below the significance threshold of 0.05 are displayed in bold.

Abbreviations: CI = confidence interval; FEV1 = forced expiratory volume in 1 second; FVC = forced vital capacity; IVW =invariance weighted; MR-PRESSO = MR-Pleiotropy Residual Sum and Outlier; MR.RAPS = MR-robust adjusted profile scores.
