# Supplementary material for: Causal effect of iron status on lung function: A Mendelian randomization study
Source: Front Nutr. 2022 Dec 15;9:1025212. doi: 10.3389/fnut.2022.1025212 (PMC9798299; doi:10.3389/fnut.2022.1025212)

Causal Effect of Iron Status on Lung Function: A Mendelian Randomization Study

Zhimin Yu, Chengkai Xu, Chenggang Fang, Fangfang Zhang

Online Supplement

Supplementary Figure S1. Regression lines of MR tests from iron status on FEV1 based on conservative genetic instruments: (A) iron; (B) log10 ferritin; (C) transferrin saturation; (D) transferrin.

Supplementary Figure S2. Regression lines of MR tests from iron status on FVC based on conservative genetic instruments: (A) iron; (B) log10 ferritin; (C) transferrin saturation; (D) transferrin.

Supplementary Figure S3. Regression lines of MR tests from iron status on FEV1/FVC ratio based on conservative genetic instruments: (A) iron; (B) log10 ferritin; (C) transferrin saturation; (D) transferrin.

Supplementary Figure S4. Regression lines of MR tests from iron status on FEV1 based on liberal genetic instruments: (A) iron; (B) log10 ferritin; (C) transferrin saturation; (D) transferrin.

Supplementary Figure S5. Regression lines of MR tests from iron status on FVC based on liberal genetic instruments: (A) iron; (B) log10 ferritin; (C) transferrin saturation; (D) transferrin.

Supplementary Figure S6. Regression lines of MR tests from iron status on FEV1/FVC ratio based on liberal genetic instruments: (A) iron; (B) log10 ferritin; (C) transferrin saturation; (D) transferrin.

Supplementary Figure S7. Forrest plot of single SNP from iron status on FEV1 based on conservative genetic instruments: (A) iron; (B) log10 ferritin; (C) transferrin saturation; (D) transferrin.

Supplementary Figure S8. Forrest plot of single SNP from iron status on FVC based on conservative genetic instruments: (A) iron; (B) log10 ferritin; (C) transferrin saturation; (D) transferrin.

Supplementary Figure S9. Forrest plot of single SNP from iron status on FEV1/FVC based on conservative genetic instruments: (A) iron; (B) log10 ferritin; (C) transferrin saturation; (D) transferrin.

Supplementary Figure S10. Forrest plot of single SNP from iron status on FEV1 based on liberal genetic instruments: (A) iron; (B) log10 ferritin; (C) transferrin saturation; (D) transferrin.

Supplementary Figure S11. Forrest plot of single SNP from iron status on FVC based on liberal genetic instruments: (A) iron; (B) log10 ferritin; (C) transferrin saturation; (D) transferrin.

Supplementary Figure S12. Forrest plot of single SNP from iron status on FEV1/FVC based on liberal genetic instruments: (A) iron; (B) log10 ferritin; (C) transferrin saturation; (D) transferrin.

Supplementary Figure S1. Regression lines of MR tests from iron status on FEV1 based on conservative genetic instruments: (A) iron; (B) log10 ferritin; (C) transferrin saturation; (D) transferrin.

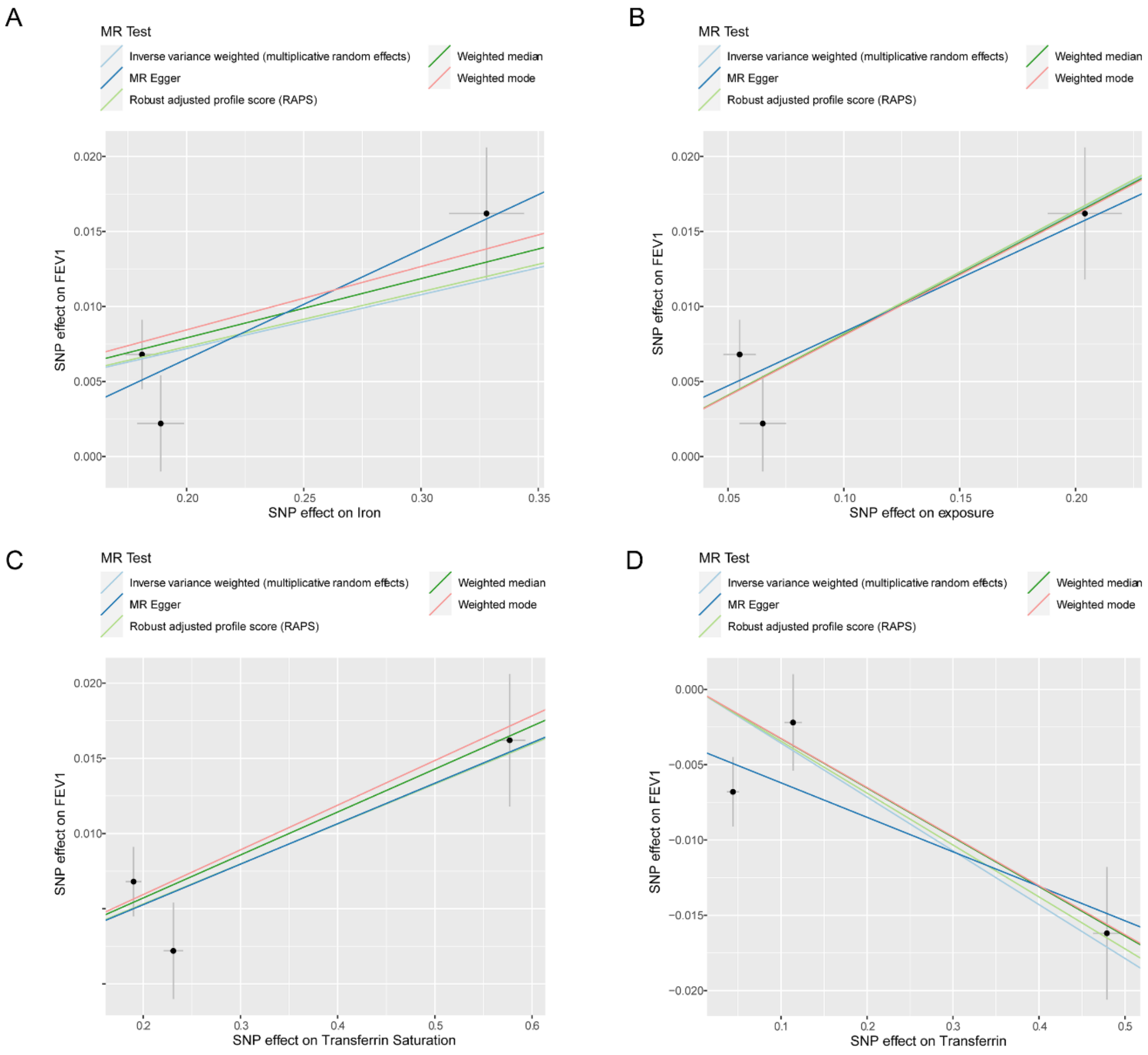

Supplementary Figure S2. Regression lines of MR tests from iron status on FVC based on conservative genetic instruments: (A) iron; (B) log10 ferritin; (C) transferrin saturation; (D) transferrin.

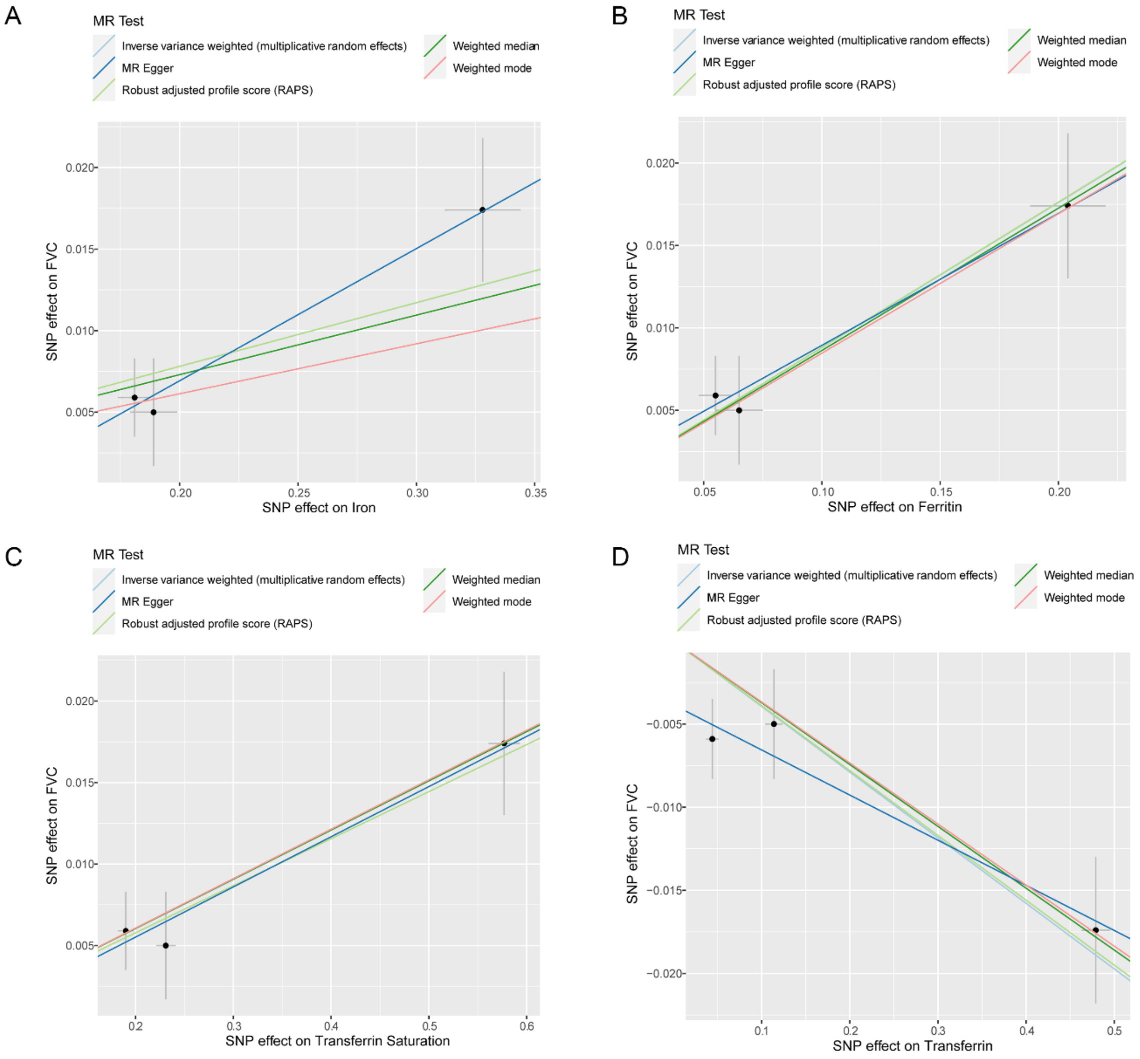

Supplementary Figure S3. Regression lines of MR tests from iron status on FEV1/FVC ratio based on conservative genetic instruments: (A) iron; (B) log10 ferritin; (C) transferrin saturation; (D) transferrin.

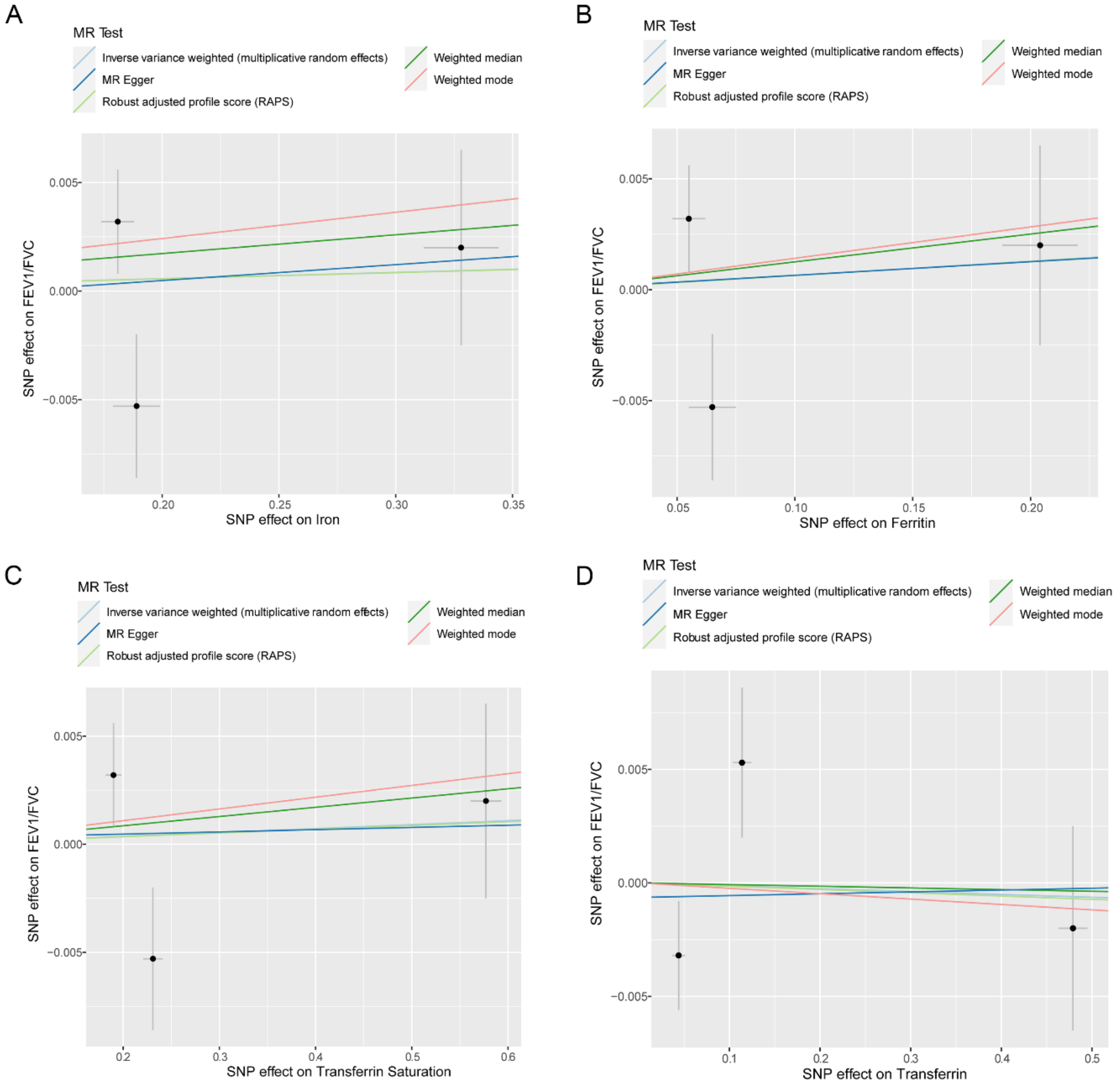

Supplementary Figure S4. Regression lines of MR tests from iron status on FEV1 based on liberal genetic instruments: (A) iron; (B) log10 ferritin; (C) transferrin saturation; (D) transferrin.

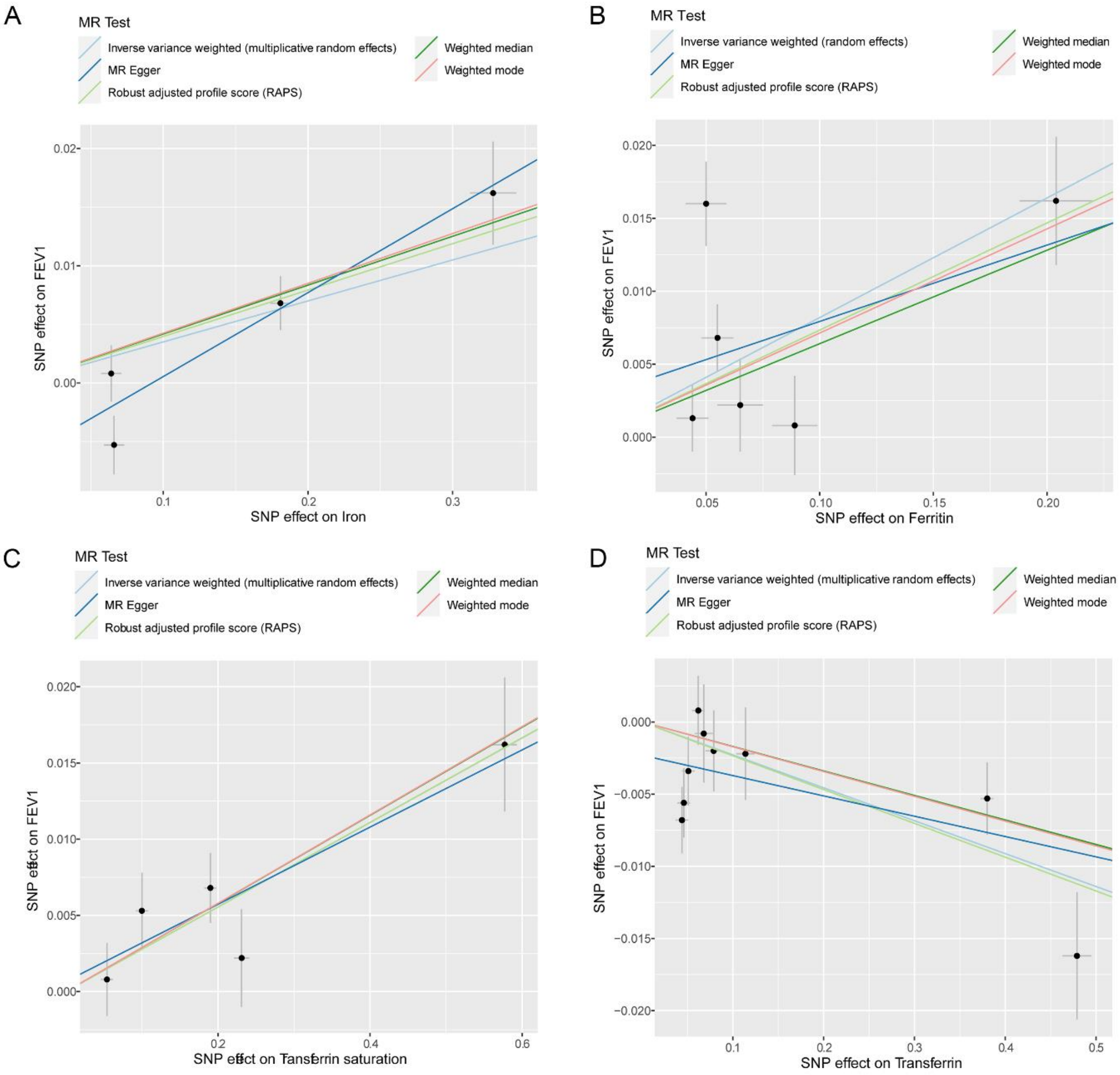

Supplementary Figure S5. Regression lines of MR tests from iron status on FVC based on liberal genetic instruments: (A) iron; (B) log10 ferritin; (C) transferrin saturation; (D) transferrin.

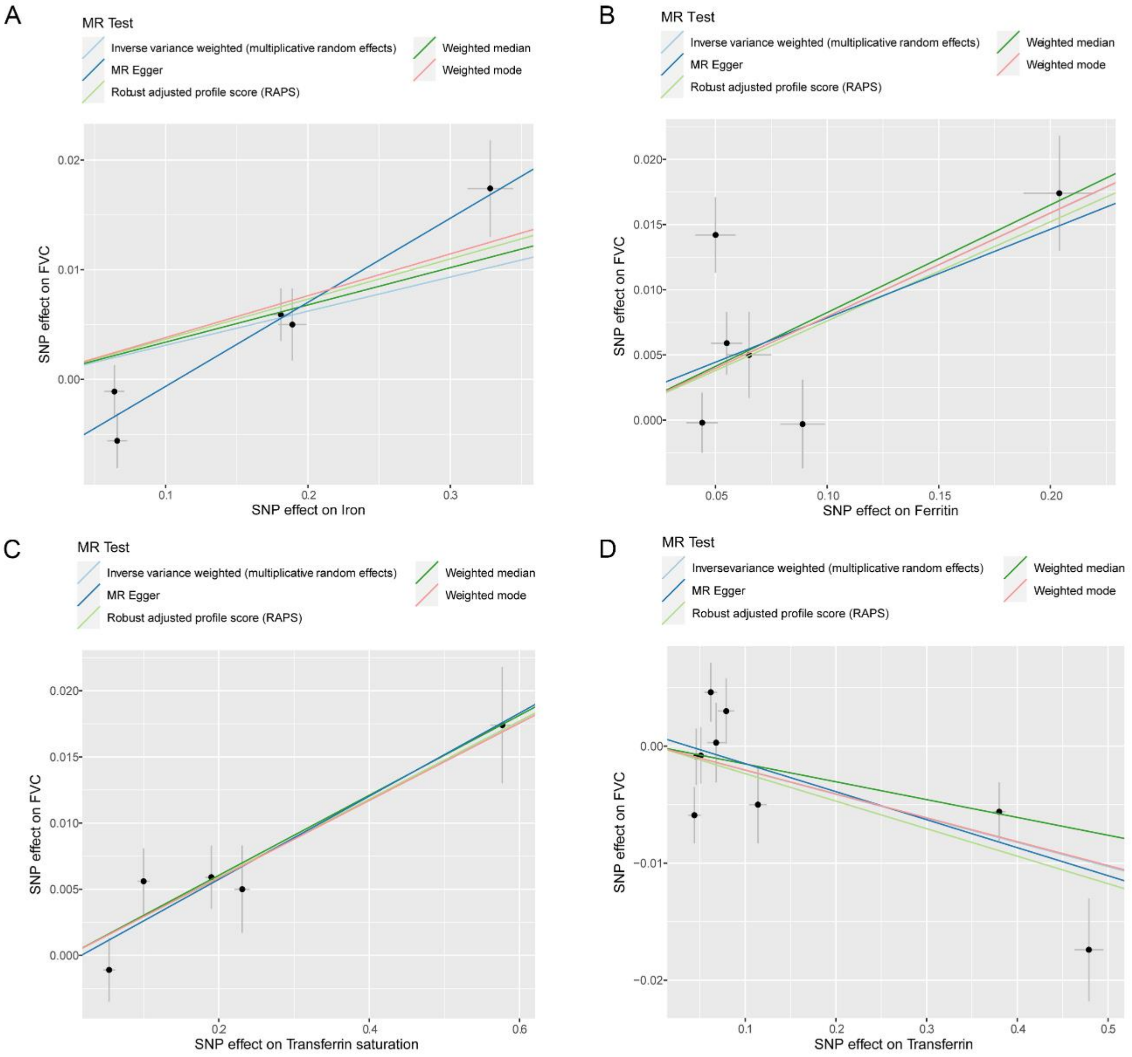

Supplementary Figure S6. Regression lines of MR tests from iron status on FEV1/FVC ratio based on liberal genetic instruments: (A) iron; (B) log10 ferritin; (C) transferrin saturation; (D) transferrin.

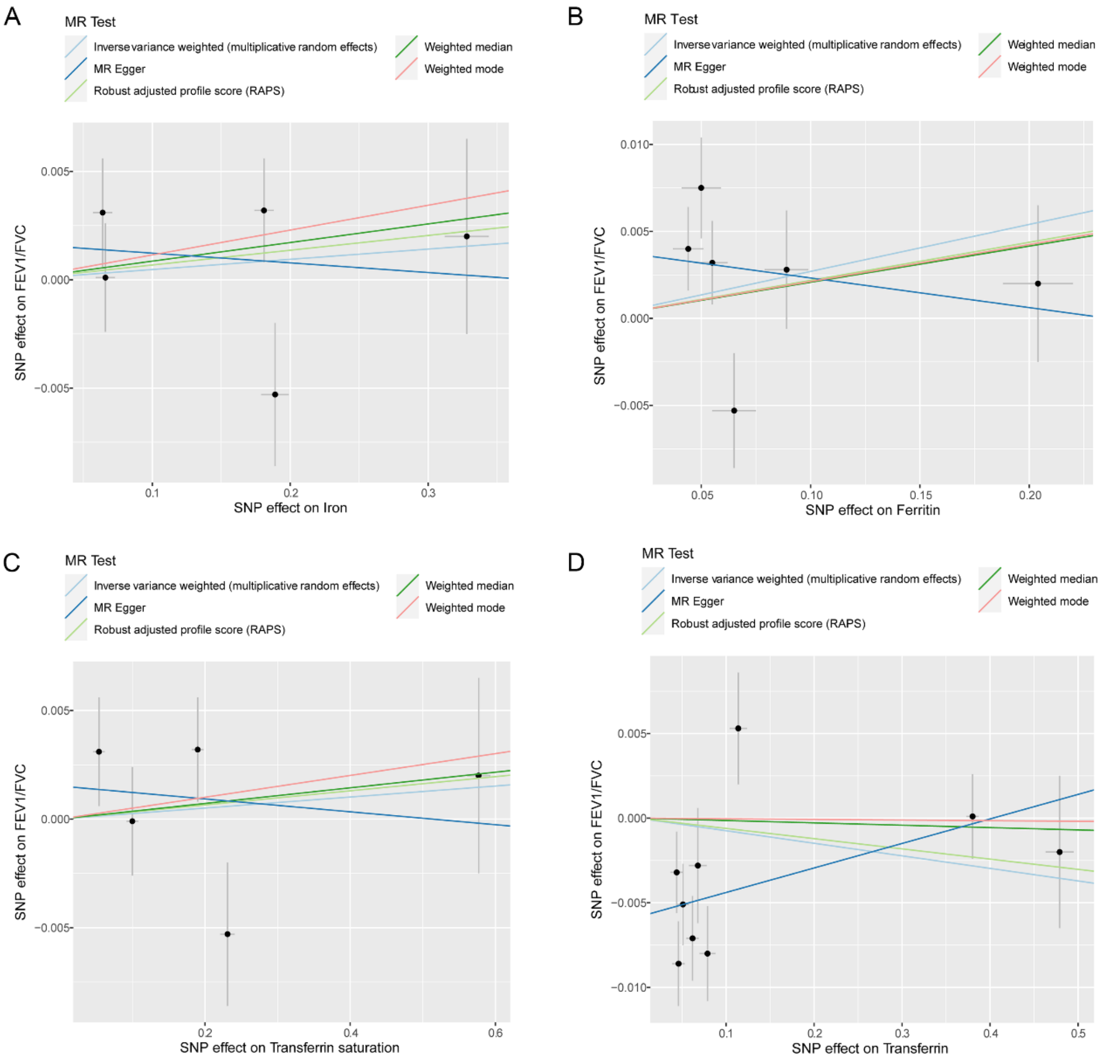

Supplementary Figure S7. Forrest plot of single SNP from iron status on FEV1 based on conservative genetic instruments: (A) iron; (B) log10 ferritin; (C) transferrin saturation; (D) transferrin.

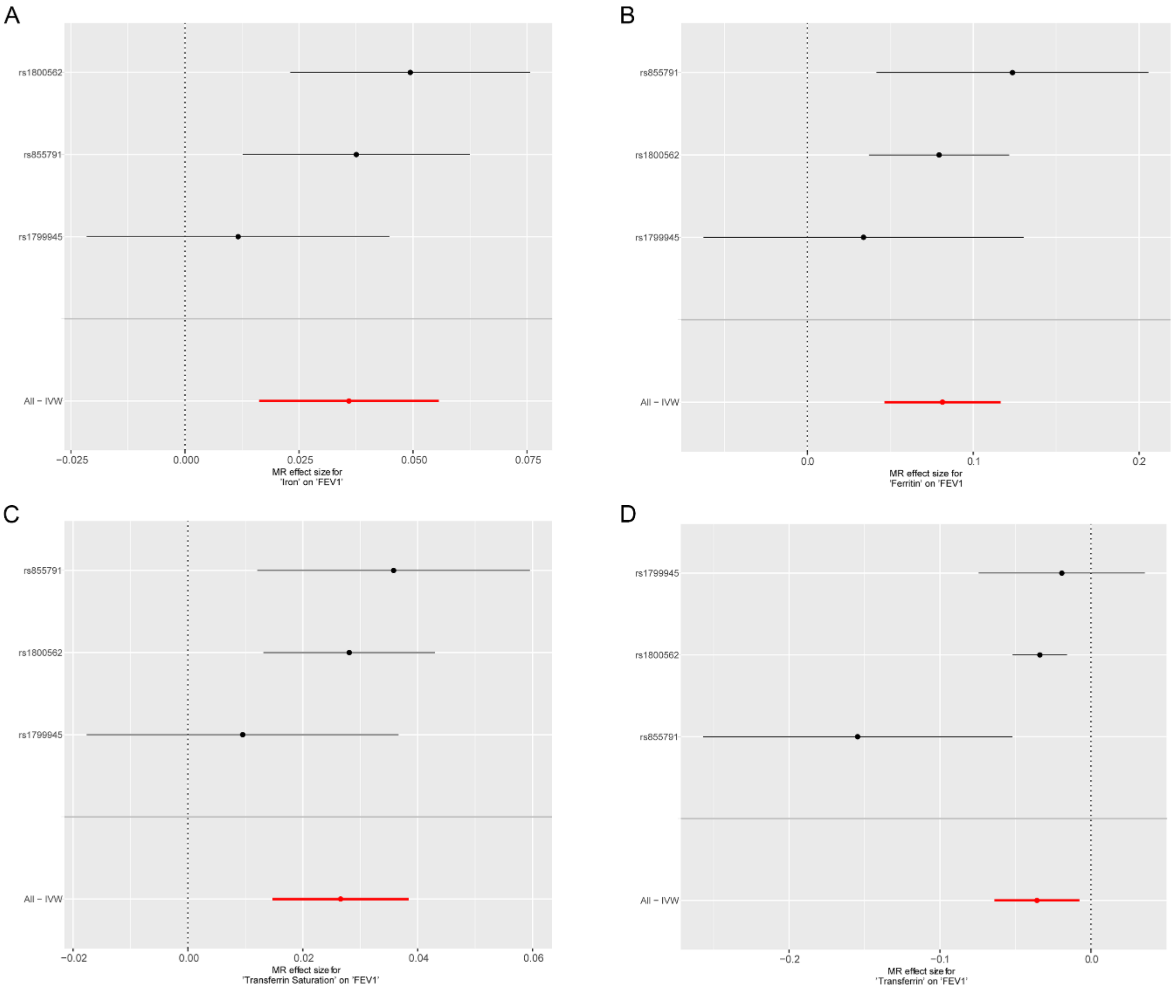

Supplementary Figure S8. Forrest plot of single SNP from iron status on FVC based on conservative genetic instruments: (A) iron; (B) log10 ferritin; (C) transferrin saturation; (D) transferrin.

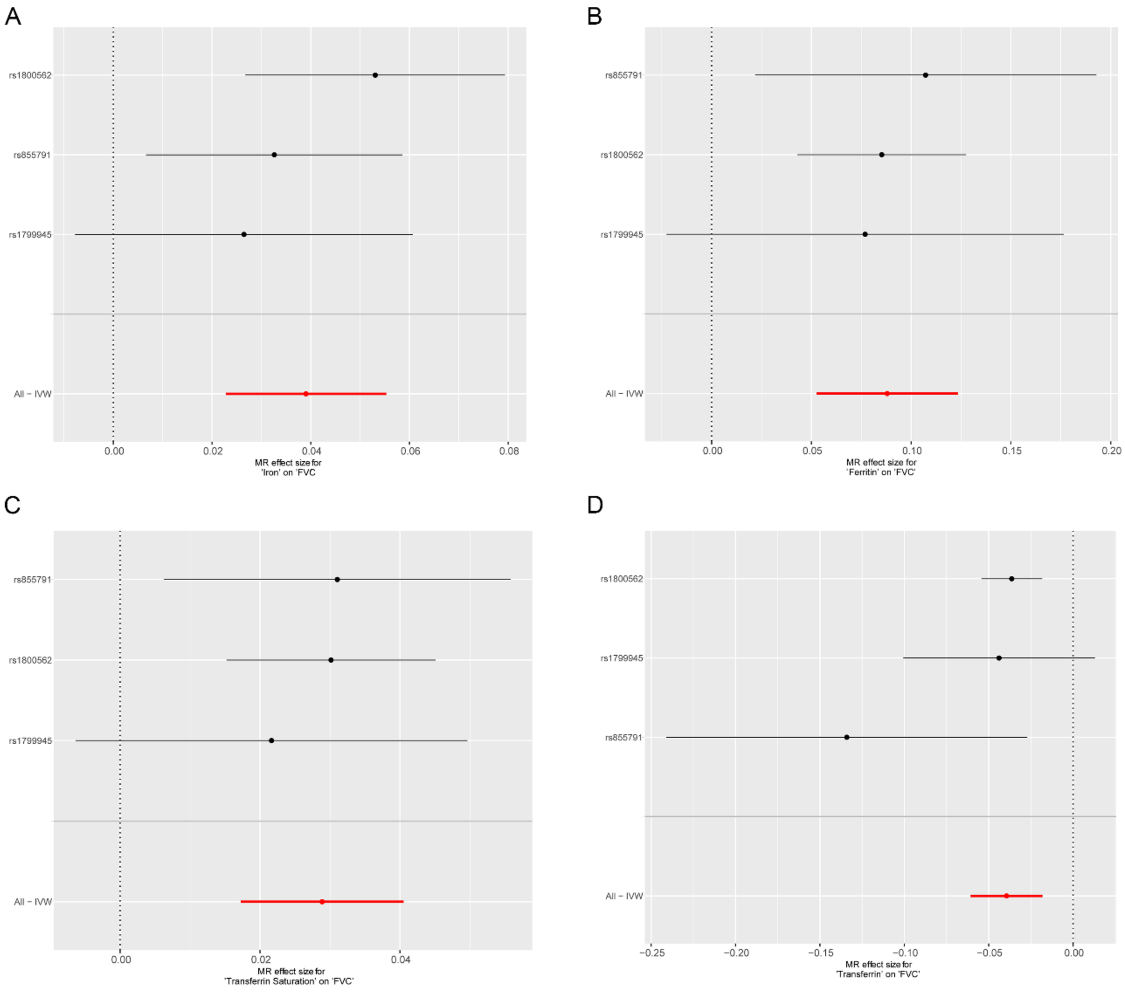

Supplementary Figure S9. Forrest plot of single SNP from iron status on FEV1/FVC based on conservative genetic instruments: (A) iron; (B) log10 ferritin; (C) transferrin saturation; (D) transferrin.

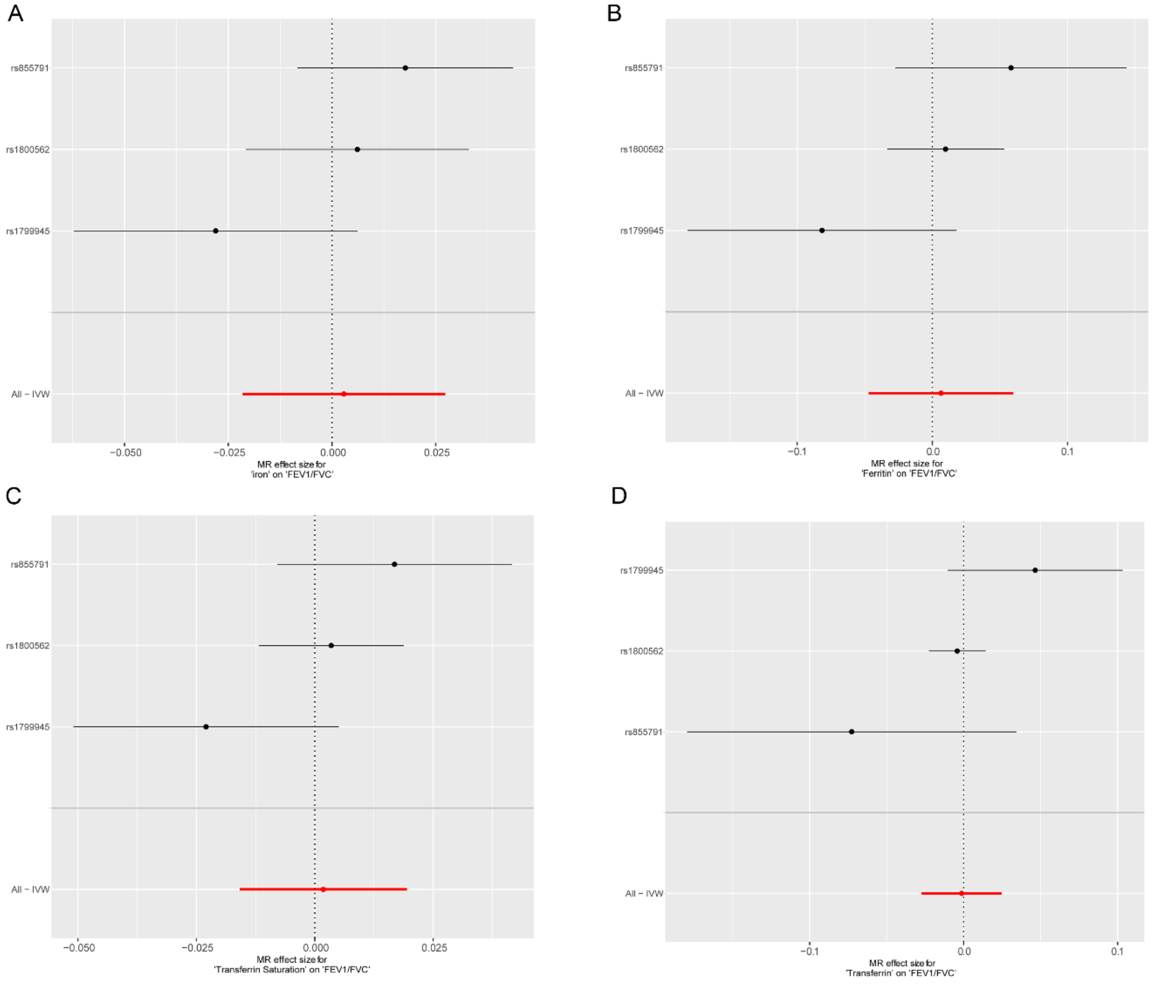

Supplementary Figure S10. Forrest plot of single SNP from iron status on FEV1 based on liberal genetic instruments: (A) iron; (B) log10 ferritin; (C) transferrin saturation; (D) transferrin.

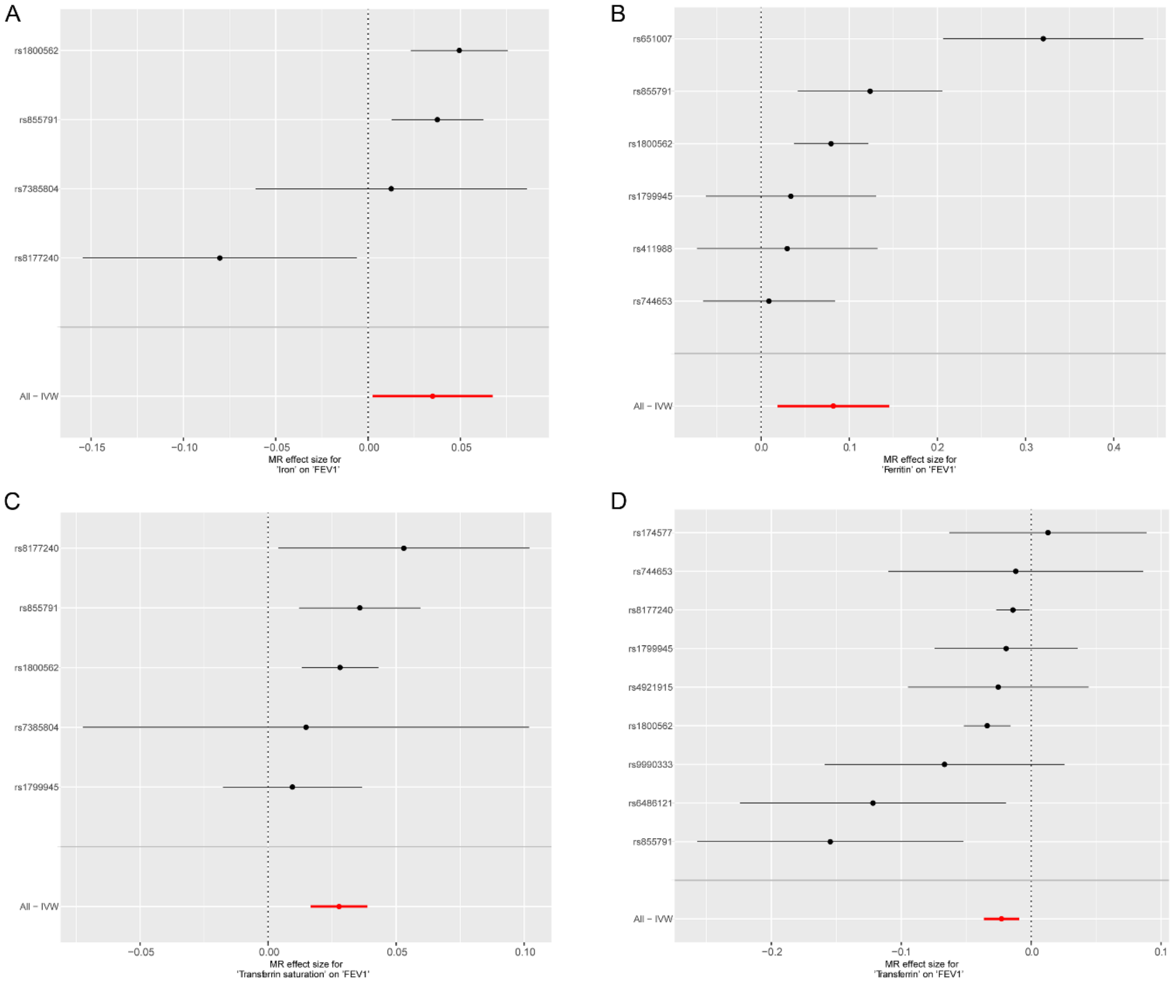

Supplementary Figure S11. Forrest plot of single SNP from iron status on FVC based on liberal genetic instruments: (A) iron; (B) log10 ferritin; (C) transferrin saturation; (D) transferrin.

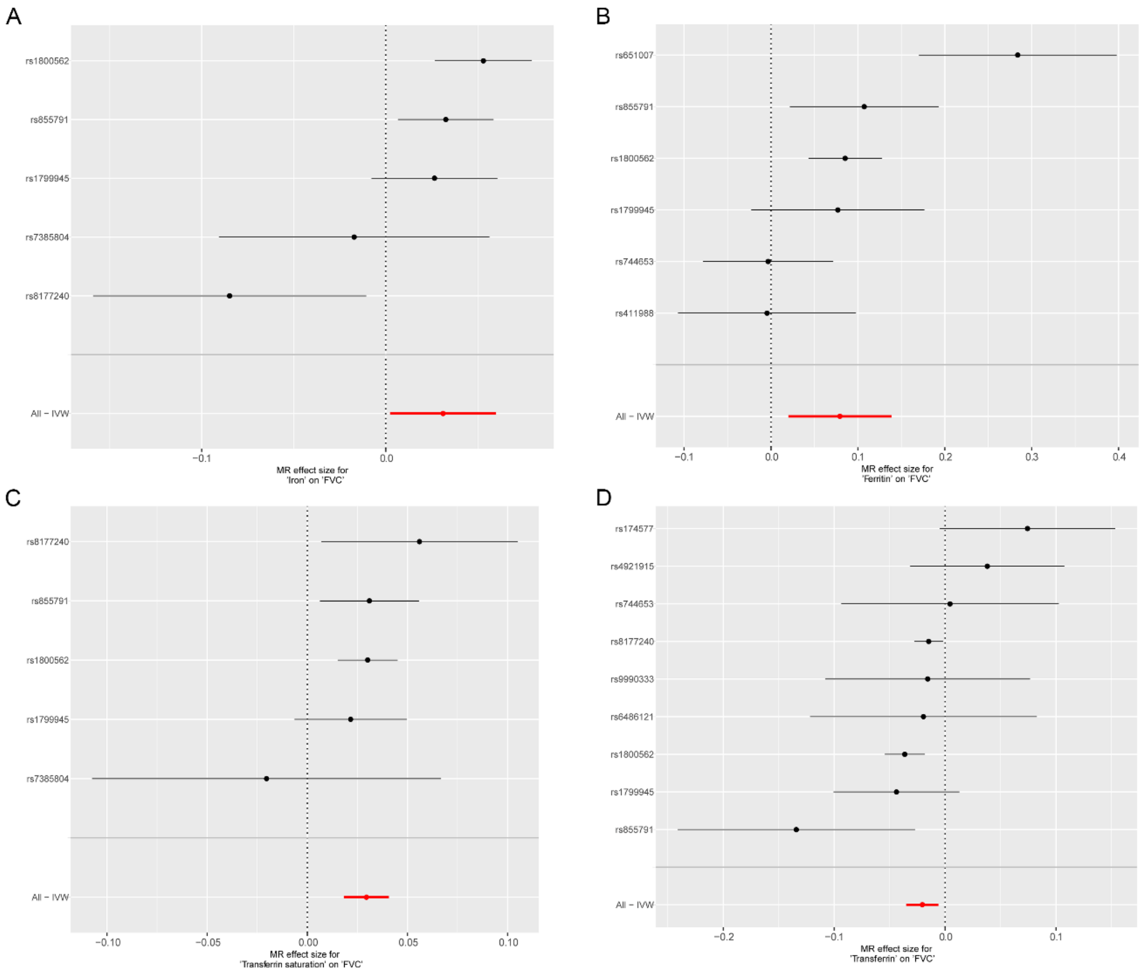

Supplementary Figure S12. Forrest plot of single SNP from iron status on FEV1/FVC based on liberal genetic instruments: (A) iron; (B) log10 ferritin; (C) transferrin saturation; (D) transferrin.

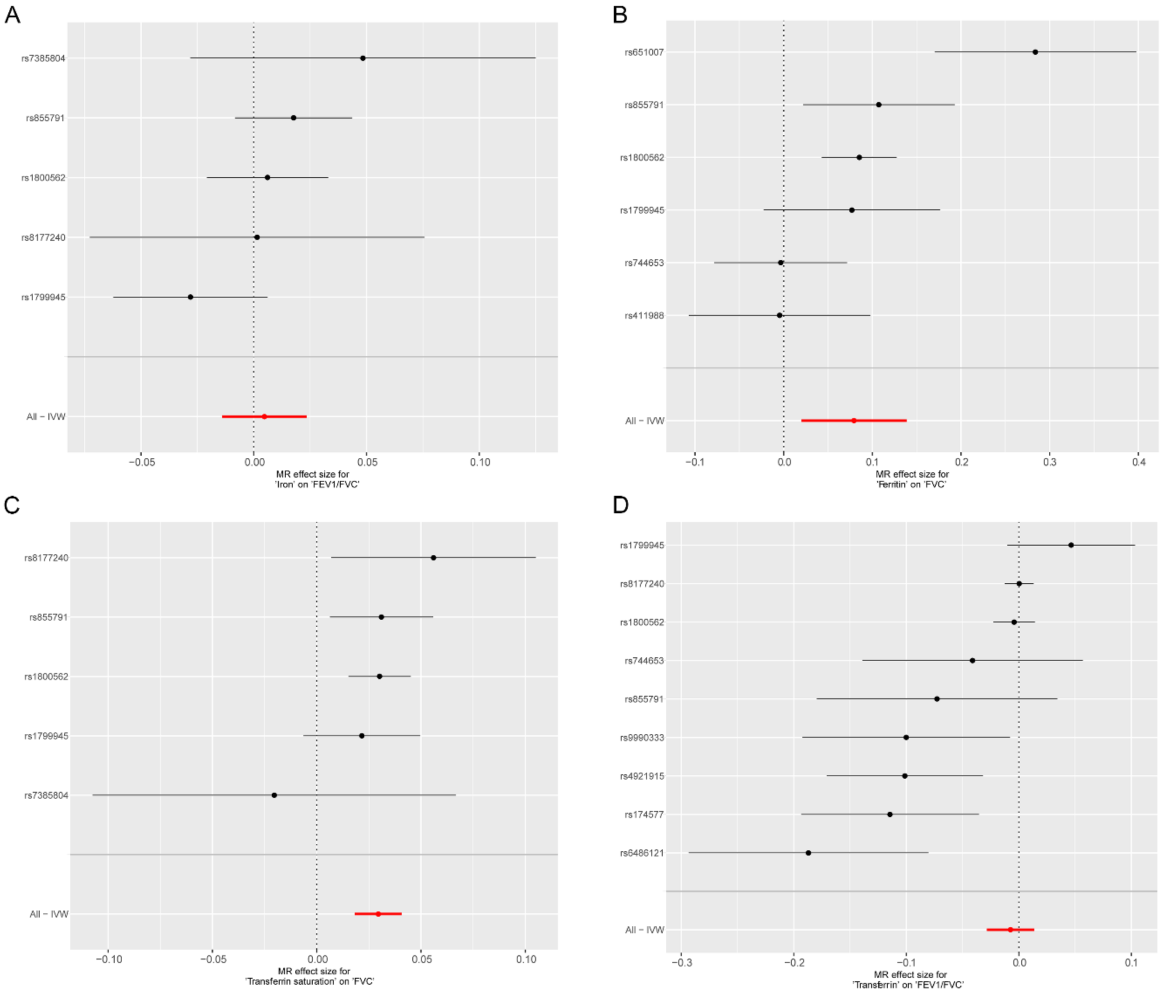

Supplement: Supplementary file 2 [file Image_1.pdf]
